# Protein palmitoylation in cancer: molecular functions and therapeutic potential

**DOI:** 10.1002/1878-0261.13308

**Published:** 2022-09-10

**Authors:** Binhui Zhou, Qianyun Hao, Yinming Liang, Eryan Kong

**Affiliations:** ^1^ Institute of Psychiatry and Neuroscience Xinxiang Medical University China; ^2^ Laboratory of Genetic Regulators in the Immune System, Henan Collaborative Innovation Center of Molecular Diagnosis and Laboratory Medicine Xinxiang Medical University China; ^3^ Key laboratory of Carcinogenesis and Translational Research (Ministry of Education/Beijing), Department of Thoracic Oncology II Peking University Cancer Hospital & Institute Beijing China; ^4^ Henan Key Laboratory of Immunology and Targeted Therapy, School of Laboratory Medicine Xinxiang Medical University China

**Keywords:** cancer treatment, oncogene, protein S‐palmitoylation, tumor suppressor, tumorigenesis

## Abstract

Protein S‐palmitoylation (hereinafter referred to as protein palmitoylation) is a reversible lipid posttranslational modification catalyzed by the zinc finger DHHC‐type containing (ZDHHC) protein family. The reverse reaction, depalmitoylation, is catalyzed by palmitoyl‐protein thioesterases (PPTs), including acyl‐protein thioesterases (APT1/2), palmitoyl protein thioesterases (PPT1/2), or alpha/beta hydrolase domain‐containing protein 17A/B/C (ABHD17A/B/C). Proteins encoded by several oncogenes and tumor suppressors are modified by palmitoylation, which enhances the hydrophobicity of specific protein subdomains, and can confer changes in protein stability, membrane localization, protein–protein interaction, and signal transduction. The importance for protein palmitoylation in tumorigenesis has just started to be elucidated in the past decade; palmitoylation appears to affect key aspects of cancer, including cancer cell proliferation and survival, cell invasion and metastasis, and antitumor immunity. Here we review the current literature on protein palmitoylation in the various cancer types, and discuss the potential of targeting of palmitoylation enzymes or palmitoylated proteins for tumor treatment.

## Graphical abstract

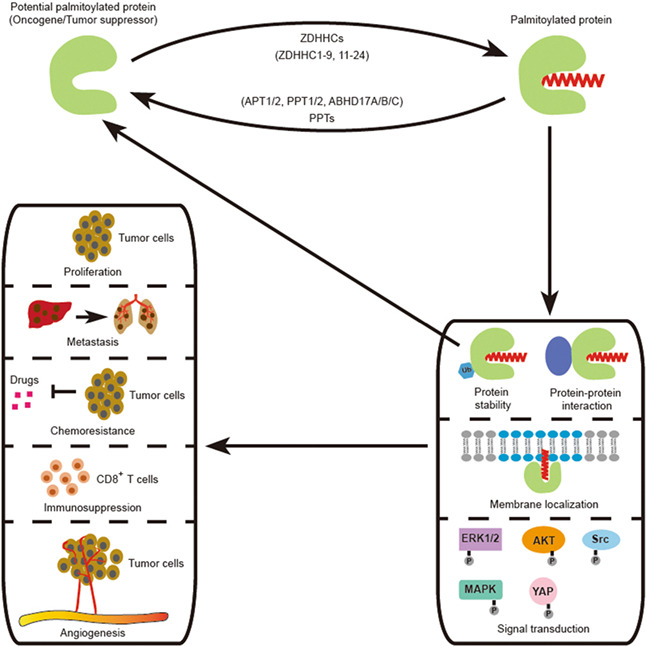

**Abbreviations**
2‐BP: 2‐Bromopalmitate
ABE: acyl‐biotin exchange
ABHD17A/B/C: alpha/beta hydrolase domain‐containing protein 17A/B/C
AML: acute myeloid leukemia
AP3D1: AP‐3 complex subunit delta‐1
APT: acyl‐protein thioesterase
AR: androgen receptor
BCR: B‐cell receptor
Cdc42: cell division cycle 42
CDCP1: CUB domain‐containing protein 1
CKAP4: cytoskeleton associated protein 4
cld7: claudin7
CLDN3: claudin 3
CRC: colorectal cancer
CSCs: cancer stem cells
Cys: cysteine
DR: death receptors
DR4: death receptor 4
DRM: detergent‐resistant membrane
EGFR: epidermal growth factor receptor
eIF3L: eukaryotic translation initiation factor 3 subunit L
EMT: epithelial to mesenchymal transition
EP300: E1A binding protein P300
EpCAM: epithelial cell adhesion molecule
ERβ: estrogen receptor β
FASN: fatty acid synthase
FBXO10: F‐box protein 10
FLT3‐ITD: internal tandem duplication within FLT3
FLT3‐ITD: internal tandem duplication within FMS‐like tyrosine kinase 3
GBM: glioblastoma
GEM: glycolipid‐enriched membrane
GPCR: G‐protein coupled receptor
HCC: hepatocellular carcinoma
HCQ: hydroxychloroquine
HGAL: human germinal center‐associated lymphoma
HHAT: hedgehog acyltransferase
HIF‐1α: hypoxia inducible factor 1 subunit alpha
HK1: hexokinase 1
IFNGR1: IFNγ receptor 1
IL6ST: interleukin 6 signal transducer
ITGβ4: integrin β4
LAT2: linker for activation of T cells family member 2
LRP6: LDL receptor related protein 6
LXR: liver X receptor
MC1R: melanocortin‐1 receptor
MCAM: melanoma cell adhesion molecule
MDH2: malate dehydrogenase 2
mtEGFR: mitochondrial EGFR
mTOR: mammalian target of rapamycin
MUC2: mucin 2
NSCLC: non‐small cell lung cancer
NTSR‐1: neurotensin receptor‐1
OMM: outer mitochondrial membrane
Palm‐B: palmostatin B
PARP: poly ADP‐ribose polymerase
PI4KIIα: phosphatidylinositol 4‐kinase IIα
PKCα: protein kinase C alpha
PLSCR1: phospholipid scramblase 1
pmEGFR: plasma membrane EGFR
PPT: palmitoyl protein thioesterase
PTMs: posttranslational modifications
PVT1: plasmacytoma variant translocation 1
RARα: retinoic acid receptor alpha
RCC: renal clear cell carcinoma
RhoU: Ras homolog family member U
SCRIB: scribble
Smad3: SMAD family member 3
TGF: transforming growth factor
TKI: tyrosine kinase inhibitor
TRAIL: tumor necrosis factor‐related apoptosis‐inducing ligand
UV: ultraviolet
VAMP3: vesicle associated membrane protein 3
ZDHHC: zinc finger DHHC‐type containing

## 1 Introduction

Tumorigenesis is characterized by persistent cell proliferation, resistance to cell death, sustained angiogenesis, and increased cell invasion and metastasis. These features are accompanied by genome instability and mutation, cellular metabolism, replicative immortality, sustained inflammation, evasion of growth suppressors, and immune suppression [1]. The above processes are often controlled by various oncogenes and tumor suppressors, many of which are modified by posttranslational modifications (PTMs), such as phosphorylation, acetylation, ubiquitination, and palmitoylation [2, 3].

**Fig. 1:**
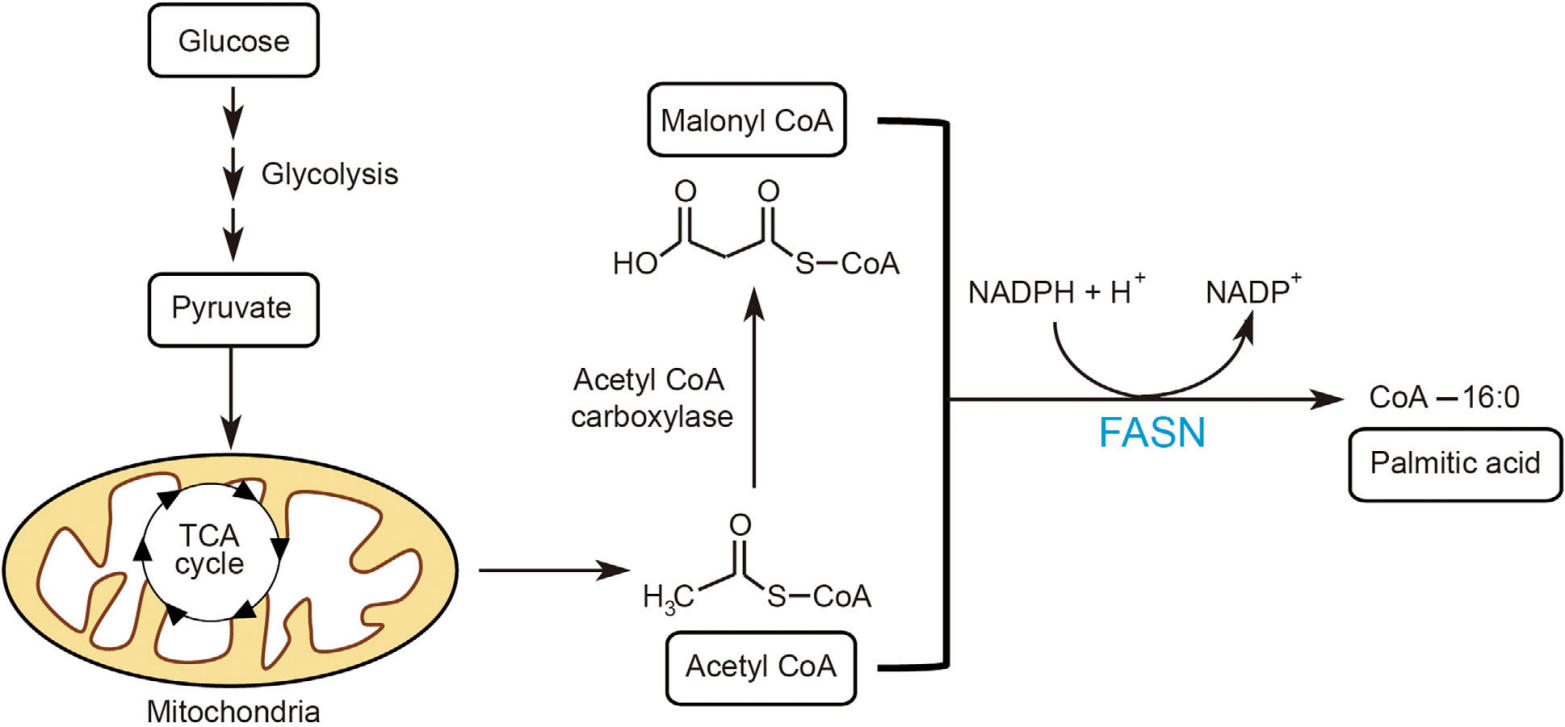
Schematic diagram of fatty acid metabolism to generate palmitic acid. Glucose is taken up by hepatocytes to generate pyruvate through glycolysis, and pyruvate is then catalyzed by pyruvate dehydrogenase in mitochondria to generate acetyl‐CoA. Next, acetyl‐CoA carboxylase catalyzes acetyl‐CoA to malonyl‐CoA. Finally, FASN exerts its multifunctional enzymatic activity to catalyze the formation of palmitic acid from acetyl‐CoA and malonyl‐CoA in multiple catalytic steps.

Protein palmitoylation is a reversible lipid modification, through which palmitate, a 16‐carbon palmitic acid, is added to a cysteine (Cys) residue via a thioester bond [4]. Palmitate is converted from fatty acids by fatty acid synthase (FASN) [5]. This process is initiated by taking up glucose in hepatocytes, followed by glycolysis to generate pyruvate; the latter is then catalyzed by pyruvate dehydrogenase to generate acetyl‐CoA in mitochondria. Acetyl‐CoA carboxylase then catalyzes acetyl‐CoA to malonyl‐CoA. Finally, FASN exerts its multifunctional enzyme activity to catalyze acetyl‐CoA and malonyl‐CoA to form palmitic acid in multiple catalytic steps (Fig. 1).

Palmitoylation is catalyzed by the zinc finger DHHC‐type containing (ZDHHC) protein family (ZDHHC1 ~ ZDHHC9, ZDHHC11 ~ ZDHHC24, also named DHHC1 ~ DHHC23), while depalmitoylation, the reverse reaction, is catalyzed by Acyl‐protein thioesterases (APT1/2), palmitoyl protein thioesterases (PPT1/2), or alpha/beta hydrolase domain‐containing protein 17A/B/C (ABHD17A/B/C) [6, 7].

**Fig. 2:**
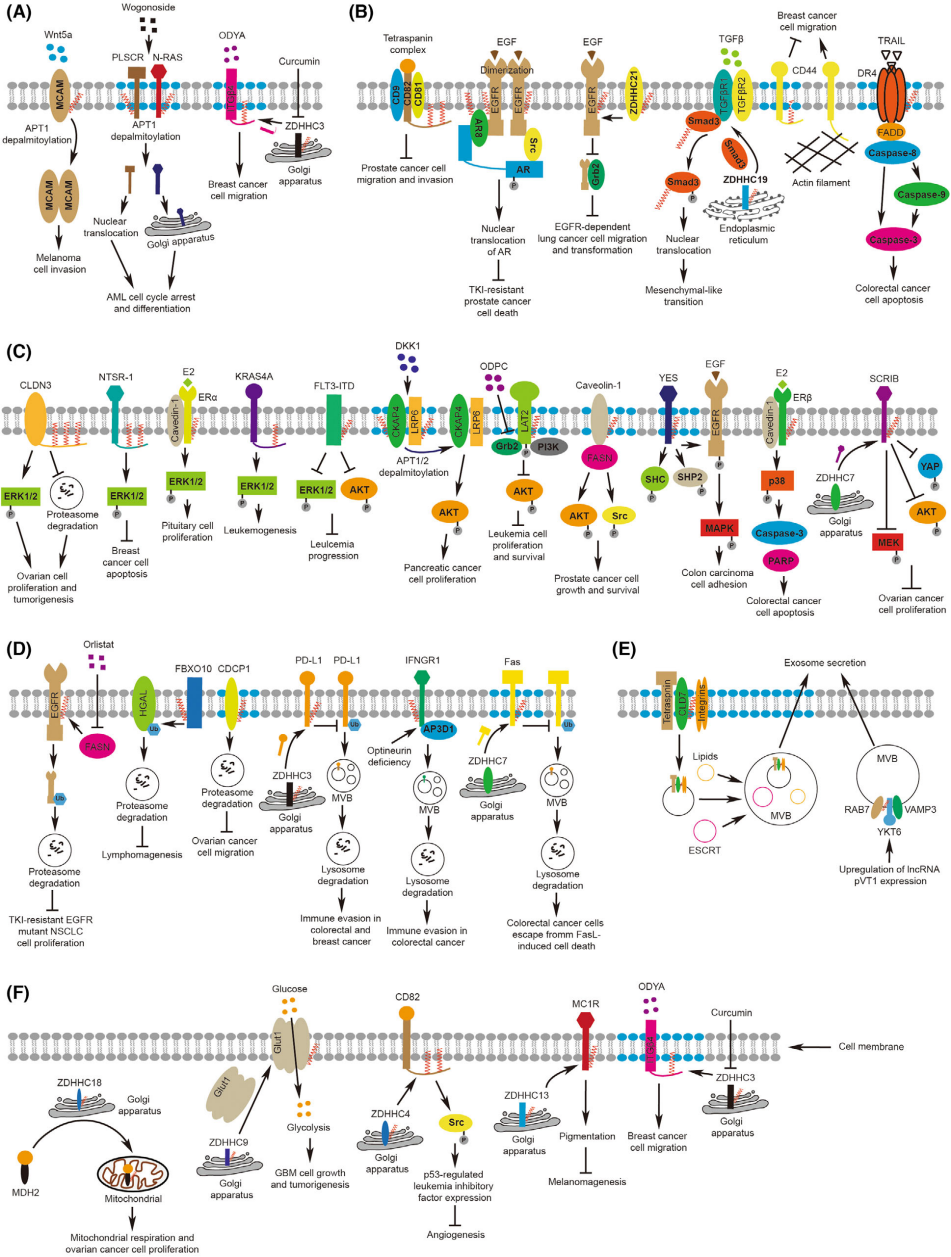
The mechanisms of the palmitoylated proteins in human cancers. (A) Palmitoylation regulates the subcellular localization of proteins. A reduced level of palmitoylation might dissociate protein from plasma membrane to cytosol or nucleus. (B) Palmitoylation regulates the protein–protein interactions. Protein palmitoylation may enhance the binding affinity of modified protein with its binding partner on/by the plasma membrane. (C) Palmitoylation modulates the activation or inhibition of signaling pathways. The palmitoylation of numerous membrane protein may activate diversified downstream signaling to control not only cell survival, proliferation, and tumor progression. (D) Palmitoylation regulates protein stability. An altered level of protein palmitoylation might induce the enhanced level of ubiquitination or internalization for protein degradation. (E) Palmitoylation regulates exosome secretion. Protein palmitoylation is also involved in the biogenesis of multiple vesicle body (MVB) and the exosome secretion mediated by MVB. (F) Palmitoylation of Glut1 regulates glucose uptake and glycolysis; CD82 palmitoylation activates Src phosphorylation and inhibits angiogenesis; the palmitoylation of MC1R augments the synthesis of pigment; curcumin targeted inhibition of ZDHHC3 and lowers the level of ITGβ4 palmitoylation and inhibits cell migration. The blue areas on the cell membrane refer to lipid rafts, and the gray areas refer to nonlipid rafts.

Biochemically, palmitoylation can significantly enhance the hydrophobicity of the modified protein in specific subdomain or subdomains, considering that a few proteins are mono‐palmitoylated and many other proteins are potentially multi‐palmitoylated. The increased level of hydrophobicity may affect varied aspects of protein features and functions; for example, it might modulate the associated affinity with membrane structure (either plasma membrane or subcellular membrane species as endoplasmic reticulum / Golgi apparatus, etc.) and thus determine the protein localization (Fig. 2A), through which the protein–protein interactions might occur or be altered (Fig. 2B) and the corresponding signaling pathways are activated or inhibited (Fig. 2C); eventually, their downstream effectors as protein stability (Fig. 2D), exosome secretion (Fig. 2E), glycolysis, mitochondria respiration, gene expression, and pigmentation (Fig. 2F) might be well regulated [8, 9, 10, 11, 12, 13, 14, 15, 16].

**Fig. 3:**
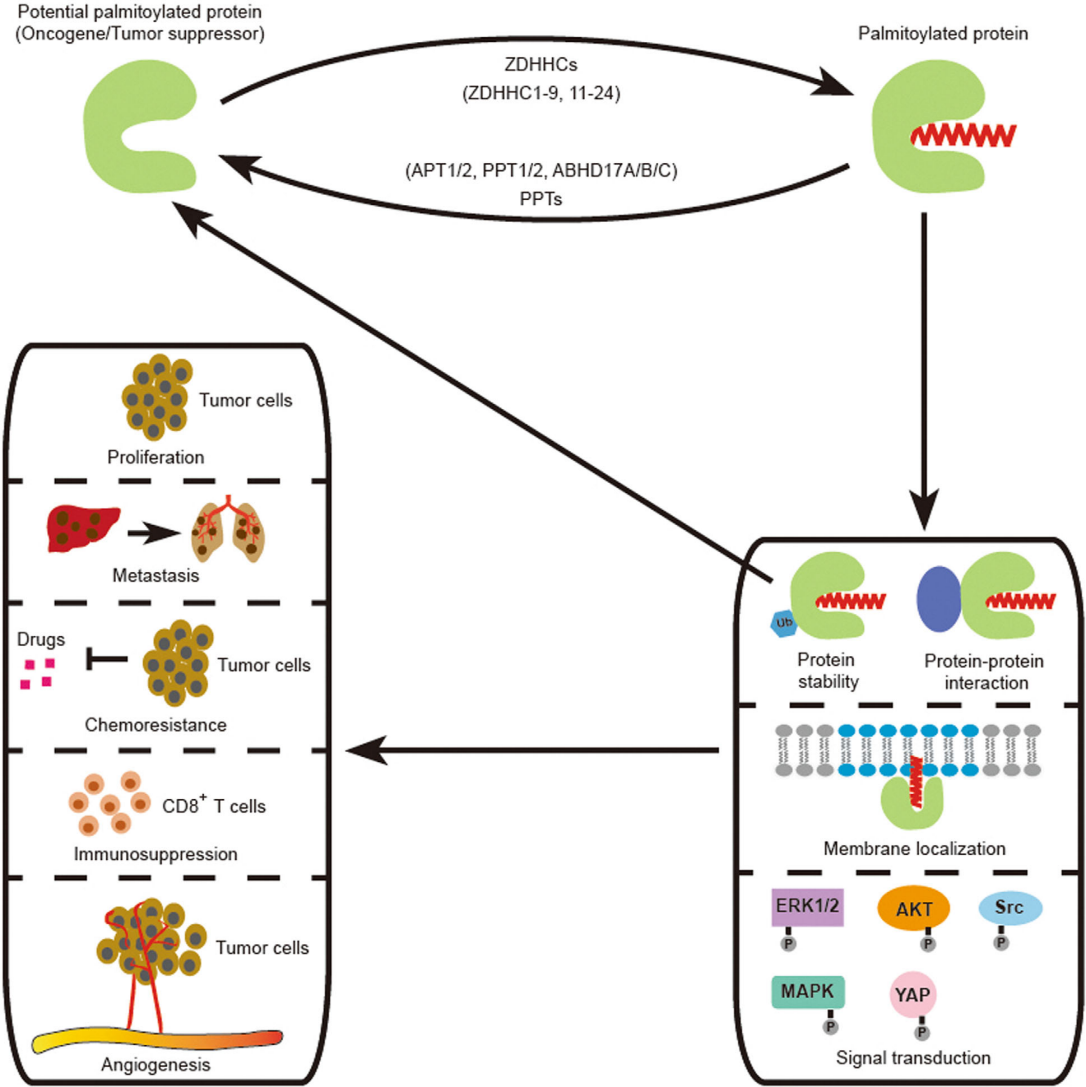
Schematic diagram of protein palmitoylation regulating tumor progression. Several oncogenes and tumor suppressors are modified by protein palmitoylation, a process that is dynamically controlled by the ZDHHC and PPT enzyme families, which add and remove palmitate, respectively. Palmitoylation affects protein stability, protein–protein interactions, membrane localization, and signaling transduction, thereby regulating tumor survival and tumor progression. Palmitoylation enzymes or palmitoylated proteins are potential targets for tumor treatment.

Numerous proteins, including key oncogenes and tumor suppressors, are palmitoylated, and their palmitoylation seems to be closely associated with tumorigenesis and tumor progression (Fig. 3) [13, 15, 17]. Despite some recent reviews summarizing the functions of ZDHHCs or PPTs in cancers [6, 18, 19, 20], a comprehensive review of this topic, particularly one focusing on the specific effects of protein palmitoylation across the various tumor types, is still lacking. Here we summarize recent findings on how protein palmitoylation affects key cancer hallmarks, including cell proliferation and survival, cell invasion and metastasis, and immune regulation in breast cancer, prostate cancer, gastrointestinal cancers, hematological cancers, melanoma, lung cancer, ovarian cancer, glioblastoma, and other tumor types. Moreover, we review tumor type‐specific patterns *versus* protein palmitoylation functions that are shared across cancers, and discuss how palmitoylation can be specifically targeted for cancer treatment in a case‐by‐case scenario.

## 2 Protein palmitoylation and breast cancer

### 2.1 Cancer cell proliferation and survival

Palmitoylation could have a direct impact on substrate activity and regulate tumor growth. It has been presented that palmitoylation of phosphatidylinositol 4‐kinase IIα (PI4KIIα) simply accelerates tumor growth in mice by regulating its catalytic activity and subcellular localization [21, 22] (Fig. 2, Table 1), and importantly, a small‐molecule inhibitor of PI‐273 that targets the palmitoylation insertion and activation loop of human PI4KIIα exhibits a significant inhibitory effect on breast cancer cell growth *in vitro* and *in vivo* [23] (Table 1). Similarly, palmitoylation also benefits tumor cells by inhibiting apoptosis: G‐protein coupled receptor (GPCR) neurotensin receptor‐1 (NTSR‐1) was found to be dually‐palmitoylated at Cys381 and Cys383 in MDA‐MB‐231 breast cancer cells, and mutation of the palmitoylation sites reduced the interaction between NTSR‐1 and Gαq/11, and decreased the localization of NTSR‐1 to the structured membrane microdomain, where Gαq/11 is preferentially present, impaired NTS‐mediated ERK1/2 stimulation, and the ability to rescue cells from apoptosis induced by serum deprivation [24].

**Table 1:** Palmitoylation‐related proteins in various cancer types.

Type of cancers	Functional proteins	Effect of palmitoylation on tumor	Agonist or antagonist of tumor phenotype	*In vivo* or *in vitro* assays	References	Palmitoylation‐related therapeutic strategy
Breast cancer	ITGβ4	ITGβ4 palmitoylation promotes invasive cancer cell migration	Agonist against invasion and growth	*In vitro* assay	[26]	LXP agonist T0901317 Knockdown of ZDHHC3 in combination with PARP inhibitor PJ‐34 Knockdown of APT1 PI4KIIα palmitoylation inhibitor PI‐273
	PI4KIIα	PI4KIIα palmitoylation accelerates tumor growth	Agonist against tumor‐induced angiogenesis	*In vivo* assay	[21, 22]	
	EGFR	EGFR palmitoylation promotes cancer cell survival	Agonist against invasion and growth	*In vitro* assay	[32]	
	NTSR‐1	NTSR‐1 palmitoylation prevents cancer cell apoptosis	Agonist against growth and metastasis	*In vitro* assay	[24]	
	PD‐L1	PD‐L1 palmitoylation enhances cancer cell immune evasion and tumor growth	Agonist against growth and immunosuppressive	*In vivo* assay	[29, 30]	
	CD82	CD82 palmitoylation prevents angiogenesis and tumor progression	Antagonist against metastasis and angiogenesis	*In vitro* and *in vivo* assay	[16]	
	CD44	CD44 palmitoylation inhibits cancer cell motility	Agonist against migration	*In vitro* assay	[25]	
	ZDHHC3	Knockdown of ZDHHC3 reduces tumor growth	Agonist against growth	*In vitro* and *in vivo* assay	[118, 119]	
	APT1	Knockdown of APT1 suppresses colony formation	Agonist against growth	*In vitro* assay	[120]	
Prostate cancer	CD82	CD82 palmitoylation inhibits cancer cell migration and invasion	Antagonist against metastasis and angiogenesis	*In vitro* assay	[41]	Knockdown of ZDHHC3 Overexpression of ZDHHC14
	Wnt1	Wnt1 palmitoylation promotes cancer cell growth rate	Agonist against growth	*In vitro* assay	[42]	
	Caveolin‐1	Palmitoylation of Cav‐1 mediates signal transduction	Both agonist and antagonist against growth and metastasis	*In vitro* assay	[43]	
	RhoU	RhoU palmitoylation attenuates cancer cell adhesion and enhances migration and invasion	Agonist against migration and invasion	*In vitro* assay	[44]	
	Fyn	Fyn palmitoylation suppresses clonogenicity	Agonist against invasion	*In vitro* and *in vivo* assay	[35]	
	AR8	AR8 palmitoylation reduces cancer cell proliferation and enhances apoptosis	Agonist against growth	*In vitro* assay	[38]	
	EGFR	EGFR palmitoylation inhibits apoptosis of TKI‐resistant cancer cells	Agonist against migration and invasion	*In vitro* assay	[93]	
	Integrin α6β4	Integrin α6β4 palmitoylation promotes cable formation	Agonist against growth, invasion, metastasis and angiogenesis	*In vitro* assay	[124]	
	eIF3L	eIF3L palmitoylation may promotes cell proliferation	Agonist against growth	*In vitro* assay	[39]	
	Rab7a and α‐tubulin	Both Rab7a and α‐tubulin palmitoylation facilitates cell proliferation	Agonist against growth	*In vitro* assay	[40]	
	ZDHHC14	Overexpression of ZDHHC14 inhibits cell viability and promotes cancer cell apoptosis	Antagonist against growth	*In vitro* and *in vivo* assay	[125]	
Colorectal cancer	ERβ	ERβ palmitoylation promotes cancer cell apoptosis	Antagonist against growth	*In vitro* assay	[47, 48]	HHAT inhibitor RU‐SKI 43 Dual MEK‐PI3K inhibitor Overexpression of ZDHHC9
	Fas	Fas palmitoylation promotes cell death	Agonist against cell death	*In vitro* assay	[49]	
	Wnt2B	Wnt2B palmitoylation level is negatively correlated with tumor malignancy	Agonist against growth	*In vitro* assay	[51]	
	YES	YES palmitoylation inhibits cell adhesion, enhances cancer cell growth and invasion	Agonist against survival, invasion, extravasation and metastasis	*In vitro* assay	[52]	
	PD‐L1	PD‐L1 palmitoylation suppresses antitumor immunity and promotes tumor growth	Antagonist against antitumor immunity and growth	*In vitro* and *in vivo* assay	[17]	
	IFNGR1	IFNGR1 palmitoylation promotes tumorigenesis	Agonist against cell apoptosis	*In vitro* and *in vivo* assay	[54]	
	DR4	DR4 palmitoylation enhances cancer cell apoptosis	Antagonist against cell apoptosis	*In vitro* assay	[47, 48]	
	Cld7	Cld7 palmitoylation promotes cancer cell motility and invasiveness	Agonist against invasion	*In vitro* and *in vivo* assay	[12]	
	MUC2	MUC2 palmitoylation facilitates tumor growth	Agonist against growth	*In vitro* and *in vivo* assay	[127]	
	ZDHHC9	Overexpression of ZDHHC9 suppresses cancer cell proliferation	Agonist against growth	*In vitro* assay	[128]	
Hematological cancers	NRAS	NRAS palmitoylation boosts leukemogenesis	Agonist against growth	*In vitro* assay	[56]	Palmitoylation inhibitor 2‐BP Depalmitoylation inhibitor Palm‐B in combination with FLT3 inhibitor gilteritinib Knockdown of APT1 and APT2 with siRNA or pharmacological inhibition of APT1 and APT2 using Palm‐B ABHD17A/B/C inhibitor ABD957
	KRAS4A	KRAS4A palmitoylation promotes leukemia progression	Agonist against growth	*In vitro* and *in vivo* assay	[59]	
	CD82	CD82 palmitoylation facilitates leukemia development	Antagonist against metastasis and angiogenesis	*In vitro* assay	[64, 65]	
	FBXO10	FBXO10 palmitoylation hinders lymphomagenesis	Agonist against cell apoptosis	*In vitro* assay	[63]	
	FLT3‐ITD	FLT3‐ITD palmitoylation inhibits leukemia cell growth	Agonist against growth	*In vitro* and *in vivo* assay	[60]	
	LAT2	LAT2 palmitoylation increases leukemic cell proliferation	Agonist against growth	*In vitro* assay	[62]	
Melanoma	MC1R	MC1R palmitoylation prevents melanomagenesis	Antagonist against growth	*In vitro* and *in vivo* assay	[15, 69]	C8 alkyl cysteine PPT1 inhibitor HCQ or DC661 Palmitoylated antigenic peptide
	MCAM	MCAM palmitoylation diminishes cell invasion	Agonist against invasion	*In vitro* and *in vivo* assay	[70, 71]	
	NRAS	NRAS palmitoylation drives tumor progression	Agonist against growth	*In vitro* and *in vivo* assay	[72, 73]	
	PPT1	Inhibition of PPT1 enhances antitumor efficacy of anti‐PD‐1 antibody	Agonist against growth	*In vitro* and *in vivo* assay	[133]	
Pancreatic cancer	CKAP4	CKAP4 palmitoylation promotes cancer cell proliferation	Agonist against growth	*In vitro* and *in vivo* assay	[79]	PPT1 inhibitor DQ661
	KRAS4A	KRAS4A palmitoylation impairs glucose consumption in cancer cells	Agonist against growth	*In vitro* assay	[80]	
	YKT6 and VAMP3	YKT6 and VAMP3 palmitoylation enhances exosome secretion and tumorigenesis	Agonist against growth	*In vitro* assay	[81]	
Non‐small cell lung cancer	EGFR	EGFR palmitoylation decreases cancer cell survival and migration	Agonist against survival and migration	*In vitro* assay	[83, 84, 85]	Knockdown of ZDHHC5 Knockdown of APT1
	ZDHHC5	Knockdown of ZDHHC5 inhibits cancer cell proliferation, invasion and colony formation, as well as hinders tumor xenograft formation	Agonist against growth and invasion	*In vitro* and *in vivo* assay	[136]	
	APT1	Silencing APT1 reduces cell proliferation, migration, and invasion	Agonist against invasion and metastasis	*In vitro* assay	[137]	
Glioblastoma	SLC1A3	SLC1A3 palmitoylation augments glutamate uptake	Agonist against growth and invasion	*In vitro* assay	[87]	Substrate‐analog inhibitors (2‐BP, cerulenin or tunicamycin)
	GP130	GP130 palmitoylation favors the proliferation and self‐renewal of glioblastoma stem cell	Agonist against growth	*In vitro* and *in vivo* assay	[139]	
	Smad3	Smad3 palmitoylation promotes mesenchymal‐like transition in the mesenchymal subtype of GBM	Agonist against growth	*In vitro* assay	[86]	
	GLUT1	GLUT1 palmitoylation enhances glycolysis, cell proliferation, and GBM tumorigenesis	Agonist against growth	*In vitro* and *in vivo* assay	[13]	
Ovarian cancer	CDCP1	CDCP1 palmitoylation increases cancer cell migration	Agonist against migration	*In vitro* and *in vivo* assay	[94]	FASN inhibitors TVB‐3166 and TVB‐3664 in combination with taxane
	EpCAM	EpCAM palmitoylation promotes cancer progression	Antagonist against metastasis	*In vitro* assay	[91]	
	CLDN3	CLDN3 palmitoylation inhibits carcinogenesis	Agonist against growth and metastasis	*In vitro* and *in vivo* assay	[90]	
	SCRIB	SCRIB palmitoylation suppresses HRas^V12^‐induced cell invasion	Antagonist against growth and metastasis	*In vitro* assay	[92]	
	MDH2	MDH2 palmitoylation promotes cancer cell proliferation	Antagonist against growth and invasion	*In vitro* and *in vivo* assay	[14]	
Hepatocellular carcinoma	CD44	CD44 palmitoylation facilitates cancer cell migration and metastasis	Agonist against migration and metastasis	*In vitro* and *in vivo* assay	[96]	PPT1 inhibitor GNS561 in combination with a multi‐kinase inhibitor sorafenib
	PPT1	PPT1 inhibition prevents tumor development and cell proliferation	Antagonist against growth	*In vitro* assay	[142, 143]	
Bladder carcinoma	CKAP4	CKAP4 palmitoylation blocks normal bladder epithelial cell proliferation	Antagonist against growth	*In vitro* assay	[99]	No relevant reports
Pituitary tumor	ERα	ERα palmitoylation enhances tumor cell proliferation	Agonist against growth	*In vitro* assay	[101, 102, 103]	No relevant reports
Gastric adenocarcinoma	ZDHHC2	Reduced ZDHHC2 expression is associated with lymph node metastasis	Antagonist against metastasis	*In vitro* assay	[100]	No relevant reports
Osteosarcoma	No relevant reports					Adriamycin in combination with palmitoylation inhibitor 2‐BP

### 2.2 Cancer cell invasion or metastasis

Palmitoylation is also suggested to be involved in controlling metastasis in breast cancer, either promoting or inhibiting. For instance, palmitoylation of CD44 at Cys286 and Cys295 inhibited cell migration by promoting the lipid raft affiliation, and abrogating palmitoylation by mutating the palmitoylation site reduced CD44 raft localization, increased CD44‐ezrin interaction, and improved invasive MDA‐MB‐231 cell migration [25]. On the contrary, the palmitoylation of integrin β4 (ITGβ4) at cysteines Cys732, Cys736, Cys738, Cys739, and Cys742 by ZDHHC3 maintains its level in lipid rafts and promotes the invasive ability of breast cancer cells [26]. Intriguingly, curcumin, a natural polyphenol component of *Curcuma longa*, effectively inhibited breast cancer cell invasion by blocking autopalmitoylation of ZDHHC3, which regulates ITGβ4 palmitoylation [26]. As angiogenesis brings nutrients and oxygen, it is conducive to tumor cell metastasis. KAI1/CD82 is a tumor metastasis suppressor in various cancers without affecting tumor formation [27]; it is significantly downregulated in estrogen receptor (ER)‐positive breast cancer, and the ER antagonist fulvestrant was able to reverse ER‐mediated gene repression, induce significant KAI1/CD82 upregulation, and inhibit breast cancer cell proliferation and migration [28]. Interestingly, ZDHHC4‐mediated palmitoylation localizes KAI1/CD82 to the cell membrane surface and induces the production of leukemia inhibitory factor through the Src/p53 pathway [16]. This in turn inhibits angiogenic factors in pericytes and endothelial cells themselves, thereby preventing angiogenesis and tumor progression [16] (Fig. 2, Table 1). Limitations: many of these studies are performed only *in vitro* (cancer cell lines); more evidence from xenografts or *in vivo* tumor models will strengthen these claims.

### 2.3 Cancer inflammation or tumor immunity

In connection with immune‐response, ZDHHC9‐mediated palmitoylation serves a pivotal role in regulating the stability of programmed cell death 1 ligand 1 (PD‐L1), which binds to programmed cell death protein 1 (PD‐1) on T‐cells and transmits immunosuppressive signals [29]. Interestingly, the inhibition of PD‐L1 palmitoylation by mutating the palmitoylation site or knocking down of palmitoylation enzyme with shRNA sensitized breast cancer cell lines to T‐cell killing, thereby suppressing tumor growth in mice [30].

### 2.4 Cancer metabolism

Recent studies have shown that *de novo* synthesized fatty acids, including palmitate, benefit tumor cells [31]. Signaling through the plasma membrane epidermal growth factor receptor (EGFR, pmEGFR) and mitochondrial EGFR (mtEGFR) is often overactivated in cancer, and EGF‐activated pmEGFR has been reported to increase FASN activity, and consequently *de novo* palmitate synthesis in prostate and breast cancer cell lines (pC3 and MDA‐MB‐231 cells, respectively). Increased palmitate levels then result in mtEGFR palmitoylation at Cys797 and subsequent mtEGFR activation to decrease the sensitivity of pC3 cells to tyrosine kinase inhibitors (TKI) [32].

## 3 Protein palmitoylation and prostate cancer

### 3.1 Cancer cell proliferation and survival

Many reported palmitoylated proteins related to prostate cancer are involved in regulating cell proliferation or survival. Src family kinases Src, Fyn, or Lyn act as major convergence points for numerous receptors and cell‐autonomous signaling pathways, resulting in enhanced cell proliferation, and metastatic potential during cancer progression [33, 34]. Palmitoylated Src and Fyn have similar roles in regulation of clone formation of prostate cells. Palmitoylation of mutated Src (Serine 3 and Serine 6 were mutated to Cys) inhibited Src activation and led to suppressed clonogenicity, whereas a lack of palmitoylation in mutated Fyn (where Cys3 and Cys6 were mutated to Serine) showed dramatically increased clonogenicity [35]. Yet these studies did not elucidate the underlying molecular mechanisms downstream of Src or Fyn palmitoylation for their functions. Androgen receptor (AR) has a vital role in prostate cancer [36]. When the hormonal ligands testosterone and 5‐dihydrotestosterone bind to AR, it dissociates from accessory proteins and transfers to the nucleus, triggering the expression of genes involved in cell proliferation and evasion of apoptosis, thereby boosting prostate cancer development [37]. A novel AR splice variant, AR8, was found to be localized to the plasma membrane via palmitoylation at Cys588 and Cys560, and overexpression of AR8 reduced prostate cancer cell proliferation and enhanced apoptosis in androgen‐depleted culture conditions [38] (Fig. 2, Table 1). Although the molecular machinery in reaction to AR signaling still remains in mystery, another study did briefly suggest that upon androgen treatment, the levels of eukaryotic translation initiation factor 3 subunit L (eIF3L), Rab7a, and α‐tubulin palmitoylation are elevated, which are required for the proliferation of prostate LNCaP cells [39, 40].

### 3.2 Cancer cell invasion or metastasis

A study published in 2004 showed that the cancer metastasis suppressor KAI1/CD82 was palmitoylated at multiple sites (Cys5, Cys74, Cys83, Cys251, and Cys253) to inhibit migration and invasion of prostate cancer cells; and palmitoylation deficiency by mutating palmitoylation sites resulted in the loss of this inhibitory effect [41]. This study demonstrated a role for protein palmitoylation in the inhibition of prostate cancer progression.

#### 3.2.1 Cancer metabolism

In immortalized prostate epithelial cells, overexpression of FASN activated β‐catenin via Wnt1 palmitoylation, and subsequently activated ligand‐independent AR [42]. Furthermore, FASN was reported to be transiently associated with lipid rafts, where it interacted with palmitoylated Caveolin‐1, thereby contributing to prostate cancer progression [43]. Therapeutically, it was demonstrated that the knockdown of FASN with shRNA reduced intracellular palmitate levels, thereby inhibiting palmitoylation of the atypical GTPase Ras homolog family member U (RhoU). RhoU palmitoylation inhibition resulted in a significant loss of cell division cycle 42 (Cdc42) expression in 1542‐CPTX primary prostate cancer cell line, ultimately increasing cell adhesion and inhibiting migration and invasion [44]. While FASN can boost the synthesis of fatty acids, including palmitate, and upregulate palmitoylation of potential different substrates, one important question here is: What are these substrates and how would these palmitoylated molecules coordinate in response to FASN signaling?

## 4 Protein palmitoylation and colorectal cancer

### 4.1 Cancer cell proliferation and survival

Palmitoylation was shown to be closely related to the progression of colorectal cancer (CRC) [45, 46]. Stimulation of 17β‐estradiol (E2) facilitates palmitoylation‐dependent membrane localization of ERβ and its binding to Caveolin‐1 and p38, thereby promoting apoptosis by p38/MAPK pathway activation in human colon adenocarcinoma DLD‐1 cells [45]. However, contradictory results were also reported that the palmitoylation of ERβ or the inhibition of p38/MAPK signaling promoted colorectal cancer cell growth [46]. Hence, the precise roles of ERβ palmitoylation and related downstream signaling cascades require further verification. Interestingly, more evidence seems to support that palmitoylation enhances apoptosis in CRC, the death receptors 4 (DR4) palmitoylation enhanced its lipid rafts localization in oxaliplatin resistance CRC cell lines, which enhanced tumor necrosis factor‐related apoptosis‐inducing ligand (TRAIL) sensitivity and bind to DR4, thereby inducing cell apoptosis [47, 48]. Likely, another death receptor, Fas (also termed CD95), was palmitoylated by ZDHHC7 at Cys199 for increasing its stability and lipid raft localization, and inhibition of Fas palmitoylation by knocking down of ZDHHC7 with siRNA promotes CRC cell lines to escape from FasL‐induced cell death [49]. Remaining questions are how the dynamicity of palmitoylation is maintained in different scenarios and why that is important for regulating apoptosis by varied mechanisms?

Wnt signaling has a key role in tumorigenesis, including CRC progression [50]. Of note, Wnt2B was shown to be palmitoylated, and Wnt2B palmitoylation alters its cellular localization, thereby indirectly influencing Wnt signaling. Furthermore, the level of Wnt2B palmitoylation in mitochondria is inversely correlated with intestinal tumorigenesis [51]. These findings implied that raising the palmitoylation levels of Wnt2B might bring beneficial effects in CRC.

### 4.2 Cancer cell invasion or metastasis

The palmitoylation of SFK family member YES plays an important role in regulating the activation of the RAS/MAPK signaling pathway. Mechanistically, the palmitoylation of YES at the SH4 domain regulates its localization in the cholesterol‐rich membrane microdomain, which augmented the phosphorylation of EGFR, SHC, and SHP2, the upstream regulators of RAS/MAPK signaling, thereby inhibiting colon carcinoma cell adhesion and promoting invasion [52]. Akin to YES palmitoylation, palmitoylated claudin7 (cld7) was also reported to regulate cell motility. Specifically, palmitoylation facilitates cld7 to localize to the glycolipid‐enriched membrane (GEM) domain, and mutating the palmitoylation site of cld7 reduces motility and invasiveness of rat pancreatic adenocarcinoma cells due to the possible mechanism that cld7 palmitoylation is involved in regulating the interactions with various components of vesicle transport machineries engaged in exosome biogenesis [12] (Fig. 2, Table 1). In agreement, another independent study indicated that tumor exosomes secreted by cancer‐initiating cells, containing GEM‐localized cld7, promote tumor cell dissemination and metastatic growth [53].

### 4.3 Cancer inflammation or tumor immunity

PD‐L1 is specifically palmitoylated at Cys272 by ZDHHC3 in human colorectal cancer cell lines; inhibiting such palmitoylation promotes ubiquitin‐mediated protein degradation of PD‐L1, thereby activating antitumor immunity and significantly suppressing tumor growth [17]. Accordingly, a competitive inhibitor (CPP‐S1 peptide) of PD‐L1 palmitoylation was developed, which reduces PD‐L1 expression in tumor cells and enhances T‐cell immunity against MC38 tumor [17]. Related to the theme of manipulating immunity by palmitoylation, IFNγ receptor 1 (IFNGR1) was reported to be palmitoylated at Cys122, which then increased its interaction with AP‐3 complex subunit delta‐1 (AP3D1) upon the removal of optineurin, resulting in palmitoylated‐IFNGR1 being sorted into lysosomes for destruction, ultimately suppressing T‐cell‐immunity and impairing immunotherapy efficacy [54] (Fig. 2, Table 1).

## 5 Protein palmitoylation and hematological cancers

Hematological cancers are commonly known as blood cancers, which usually form in the bone marrow or cells of the immune system. Although called blood cancer, hematological cancers include cancers such as a wide range of myelomas, lymphomas, and leukemias.

### 5.1 Cancer cell proliferation and survival

The RAS family members HRAS, NRAS, and KRAS were the first oncogenes identified in human cancers [55]. A 2018 study focusing on Wogonoside—a flavonoid extracted from *Scutellaria baicalensis Georgi* with antileukemic properties—reported that Wogonoside inactivates the NRAS/RAF1 signaling pathway by blocking NRAS palmitoylation in acute myeloid leukemia (AML) cells [56]. Of note, Wogonoside was also shown to promote depalmitoylation of yet another target, namely of phopospholipid scramblase 1 (PLSCR1) via targeting APT1 [56]. A second study confirmed that the association of NRAS with the plasma membrane requires palmitoylation at Cys181, and removal of palmitoylation was found to inactivate multiple signaling pathways downstream of oncogenic NRAS, ultimately suppressing leukemogenesis [57]. KRAS4A was also reported to be modified by palmitoylation at Cys180 [58]. Interestingly, leukemia could still be induced in mice expressing palmitoylation‐deficient KRAS4A, albeit with slower kinetics. Unexpectedly, simultaneous mutation of the palmitoylation site and KIKK motif of KRAS4A abolished neurogenesis [59] (Fig. 2, Table 1). Last, ZDHHC6‐mediated palmitoylation at Cys563 restrained cell surface localization of internal tandem duplication within FMS‐like tyrosine kinase 3 (FLT3‐ITD) protein, inhibited the activation of AKT and ERK, and leukemia cell growth [60].

Moreover, another study showed that the alkyl phospholipid 10‐(octyloxy) decyl‐2‐(trimethylammonium) ethyl phosphate (ODPC), which specifically induces apoptosis in leukemia cells by targeting the high cholesterol domain in cell membranes [61], lowered palmitoylation of the linker for activation of T‐cells family member 2 (LAT2) at Cys25 and Cys28 in a lipid‐raft‐enriched fraction of leukemic cells, thereby promoting LAT2 degradation by proteasome, and hence decreasing cell proliferation and enhancing cell sensitivity to ODPC, perifosine, and arsenic trioxide [62]. Antigen‐induced B‐cell receptor (BCR) activation triggers palmitoylation of the E3 ubiquitin ligase F‐box protein 10 (FBXO10) at Cys49, Cys52, Cys180, Cys430, and Cys953, which causes FBXO10 to localize on the cell membrane to ubiquitinate and degrade the human germinal center‐associated lymphoma (HGAL) protein. HGAL downregulation then prevents the uncontrolled BCR signaling that is linked to development of lymphoid hyperplasia, and lymphomagenesis [63] (Fig. 2, Table 1). The shortcomings of these studies are that they did not specify the enzymes catalyzing palmitoylation, and some conclusions lack support from palmitoylation‐site mutant protein.

### 5.2 Cancer cell invasion or metastasis

As shown above, KAI1/CD82 palmitoylation prevents tumor metastasis in both breast and prostate cancers; however, the palmitoylation of KAI1/CD82 on the membrane of AML cells promotes the development of aggressive leukemia through recruiting and stabilizing protein kinase C alpha (PKCα) in membrane clusters, and subsequently sustaining ERK signaling [64]. Conversely, inhibiting KAI1/CD82 palmitoylation by mutating the palmitoylation site dramatically impairs the formation and organization of N‐cadherin clusters, and subsequently diminishes bone marrow homing of AML [65] (Fig. 2, Table 1). The functional discrepancies of KAI1/CD82 palmitoylation in different types of tumors might cause problems if targeting KAI1/CD82 palmitoylation, or at least, one should be cautious under such circumstances.

### 5.3 Cancer inflammation or tumor immunity

It has been suggested that immune suppression is closely related to leukemogenesis, yet possibly mediated by diversified molecular machineries. Extended evidence suggests that the uptake by monocytes of AML‐derived extracellular vesicles with abundant palmitoylated proteins on their surface promotes the differentiation of myeloid‐derived suppressor cells, thereby enhancing immune escape [66] (Fig. 2, Table 1).

## 6 Protein palmitoylation and melanoma

### 6.1 Cancer cell proliferation and survival

The melanocortin‐1 receptor (MC1R) is a member of the GPCR family that is expressed on melanocytes and enhances ultraviolet (UV) tolerance when activated [67], which reduces the risk of melanoma. However, mutational inactivation of MC1R (Mc1r RHC variants) results in reddened hair color, poorer skin tanning ability in humans, and increases the risk of melanoma [68]. Interestingly, ZDHHC13 was found to mediate MC1R palmitoylation at Cys78 and Cys315, and MC1R palmitoylation is required for activation of MC1R signaling, which leads to enhanced pigmentation, UV‐induced cell cycle arrest, and control of melanomagenesis [15]. Furthermore, employing Palm‐B to pharmacologically increase palmitoylation recovers the deficiencies of Mc1r RHC variants and inhibits melanomagenesis [15]. Two years later, another study demonstrated that APT2 is the depalmitoylation enzyme of MC1R, and ML349, a selective APT2 inhibitor, could significantly increase MC1R palmitoylation and the downstream signaling that prevents UV‐induced melanomagenesis [69] (Fig. 2, Table 1).

### 6.2 Cancer cell invasion or metastasis

It was suggested that ZDHHC20 mutants in melanoma cells reduce palmitoylation of melanoma cell adhesion molecule (MCAM) and impair the MCAM ability to inhibit cell invasion, whereas Wnt5a promotes depalmitoylation of MCAM at Cys590 via phosphorylated APT1 [70]; APT1 inhibition with Palm‐B prevents Wnt5a‐induced depalmitoylation, asymmetric MCAM localization, and cell invasion [71]. Similar to leukemia, NRAS palmitoylation is required for pro‐tumorigenic NRAS signaling in melanoma, which often carries *NRAS* activating mutations [72, 73] (Fig. 2, Table 1). Unfortunately, in the past NRAS has often been deemed as “undruggable” due to the lack of hydrophobic binding pockets on the NRAS surface [74]. Fortunately, Vora *et al*. [75] developed an amphiphile‐mediated depalmitoylation (AMD) strategy using C8 alkyl cysteine, to cleave S‐palmitoyl groups from endogenous membrane‐associated NRAS protein, resulting in a significant reduction in NRAS palmitoylation, inhibition of NRAS signaling, as well as melanoma progression (Table 1).

## 7 Protein palmitoylation and pancreatic cancer

### 7.1 Cancer cell proliferation and survival

In a study by Sanders *et al*. [76], who analyzed data from 15 palmitoylation proteomics studies, pancreatic ductal adenocarcinoma emerged as one of the top 5 diseases with enrichment of proteins that are known palmitoylation targets. This implies that aberrant palmitoylation may be involved in pancreatic tumorigenesis [76]. Published studies suggested that overexpression of cytoskeleton associated protein 4 (CKAP4) or LDL receptor‐related protein 6 (LRP6) promotes pancreatic cancer progression [77, 78]. Interestingly, CKAP4 was found palmitoylated by ZDHHC2 at Cys100, and LRP6 was palmitoylated at Cys1394 and Cys1399 (with palmitoylation enzymes not yet defined). Mechanistically, palmitoylation induces localization of CKAP4 and LRP6 in the detergent‐resistant membrane (DRM) fractions, thereby activating the PI3K‐AKT pathway and promoting cell proliferation [79]. Furthermore, a 2019 study demonstrated that depalmitoylation promotes the colocalization and direct interaction of KRAS4A and hexokinase 1 (HK1) on the outer mitochondrial membrane (OMM), which in turn promotes glucose consumption. Accordingly, disruption of KRAS4A reduces glucose consumption in pancreatic cancer cells [80]. Recently, it was also revealed that upregulation of long noncoding RNA plasmacytoma variant translocation 1 (PVT1) promotes the movement of multivesicular bodies towards the plasma membrane by regulating the colocalization of palmitoylated YKT6 at Cys194 and Cys195 and vesicle‐associated membrane protein 3 (VAMP3), which then stimulates exosome secretion, and may serve a vital role in tumorigenesis [81] (Fig. 2, Table 1).

## 8 Protein palmitoylation and non‐small cell lung cancer

### 8.1 Cancer cell proliferation and survival

Non‐small cell lung cancer (NSCLC) accounts for nearly 85% of all lung cancer cases that are clinically diagnosed [82]. The link between protein palmitoylation and NSCLC was established by the finding that ZDHHC21 palmitoylates EGFR at Cys1025, Cys1034, and Cys1122 in HEK293T cells, where EGFR and ZDHHC21 were coexpressed and purified for mass spectrometry; and inhibition of ZDHHC21 with shRNA in MDA‐MB‐231 cells or expressing the palmitoylation site mutation of EGFR plasmids in NIH 3T3 cells enhanced EGFR activation and increased EGFR signaling‐dependent cell survival and migration [83]. Moreover, another independent study also showed that blocking EGFR palmitoylation resulted in inhibition of PI3K signaling by reducing the EGFR association to p85, ultimately suppressing mutant KRAS lung tumorigenesis [84]. While these studies pointed out the potential pharmaceutical value of targeting EGFR palmitoylation for treating NSCLC, interestingly, another group presented that blocking FASN with Orlistat (a reversible inhibitor of gastric and pancreatic lipases) treatment prevents EGFR palmitoylation, enhances ubiquitination and degradation of EGFR, and then inhibits NSCLC cell proliferation, and suppresses *in vivo* tumorigenesis [85] (Fig. 2, Table 1). Taken together, similar to other types of tumors, EGFR palmitoylation also plays a critical role in NSCLC progression.

## 9 Protein palmitoylation and glioblastoma

### 9.1 Cancer cell proliferation and survival

ZDHHC19‐mediated SMAD family member 3 (Smad3) palmitoylation at Cys421 enhances activation of the transforming growth factor (TGF) signaling pathway, and Smad3 interaction with E1A binding protein P300 (EP300) promotes mesenchymal‐like transition in the mesenchymal subtype of glioblastoma (GBM) [86]. Importantly, the combined inhibition of Smad3 palmitoylation (by using 2‐BP) and hypoxia inducible factor 1 subunit alpha (HIF‐1α) successfully inhibited tumor growth and increased survival rates in mice with xenografts [86] (Fig. 2, Table 1).

### 9.2 Cancer metabolism

In advanced GBM, the membrane expression and palmitoylated form of solute carrier family 1 member 3 (SLC1A3, a glutamate transporter) were dramatically downregulated, resulting in impaired glutamate uptake [87] (Fig. 2, Table 1). However, the specific palmitoylation site in SLC1A3 and the enzymes regulating SLC1A3 palmitoylation have not yet been explored.

To maintain rapid proliferation, even in the presence of oxygen, cancer cells absorb large amounts of glucose for glycolysis, a process known as the Warburg effect [88]. The glucose transport protein GLUT1 is a critical transmembrane protein to regulate glucose transport [89]. ZDHHC9‐mediated GLUT1 palmitoylation at Cys207 maintains GLUT1 localization in the cell membrane for glucose transport. Knocking out ZDHHC9 using the CRISPR/Cas9 system or mutating the GLUT1 palmitoylation site abolished palmitoylation and cell membrane distribution of GLUT1, resulting in impaired glycolysis, cell proliferation, and GBM tumorigenesis [13] (Fig. 2, Table 1).

## 10 Protein palmitoylation and ovarian cancer

### 10.1 Cancer cell proliferation and survival

ZDHHC12 mediates palmitoylation of claudin 3 (CLDN3) at Cys103, Cys106, Cys181, Cys182, and Cys184 to enhance ovarian cancer progression and, reversely, defective CLDN3 palmitoylation inhibits membrane localization and protein stability of CLDN3, with a negative impact on ovarian cancer cell growth [90]. In addition, the palmitoylation of epithelial cell adhesion molecule (EpCAM) affects the formation of a complex among EpCAM, claudin isoforms, and KAI1/CD82, which is important in ovarian cancer progression, whereas 2‐BP treatment inhibits EpCAMP‐CLDN‐KAI1/CD82 complex formation [91]. Moreover, ZDHHC18‐medited palmitoylation of malate dehydrogenase 2 (MDH2) at Cys138 is essential for mitochondrial respiration and ovarian cancer cell proliferation both *in vitro* and *in vivo*, and loss of MDH2 palmitoylation by mutating the palmitoylation site or knocking down of ZDHHC18 with shRNA inhibited the clonogenic capability of ovarian cancer cells [14]. On the contrary, ZDHHC7‐mediated palmitoylation of the tumor suppressor function of scribble (SCRIB) at Cys4 and Cys10 is important for regulating OVCAR8 cell proliferation and inhibiting the activation of YAP, MAPK, and PI3K/AKT pathways in MCF10A cells [92] (Fig. 2, Table 1). These examples strengthen the notion that palmitoylation regulates tumorigenesis in both directions.

### 10.2 Cancer cell invasion or metastasis

Palmitoylation‐dependent EGFR signaling has been described above to play an important role in the progression of breast cancer [32], prostate cancer [93], and NSCLC [83, 84, 85]. In ovarian cancer, activation of EGFR signaling prevents proteasomal degradation of CUB domain‐containing protein 1 (CDCP1) through CDCP1 palmitoylation at Cys689, Cys690, Cys772, and Cys780 and subsequent CDCP1 localization to the cell surface, where CDCP1 acts to enhance cell migration [94] (Fig. 2, Table 1).

## 11 Protein palmitoylation and other cancers

Even though hepatocellular carcinoma (HCC) is the most frequent primary liver cancer and a serious medical problem [95], there are limited studies relating protein palmitoylation to HCC progression. A report stated that high cholesterol levels enhance the lipid raft localization of CD44 in a palmitoylation‐dependent manner, and disrupts CD44‐Ezrin binding, ultimately reducing HCC cell migration and metastasis [96] (Fig. 2, Table 1). This finding is consistent with the reported role for CD44 palmitoylation in breast cancer [25].

Moreover, protein palmitoylation has been implicated in the development of renal clear cell carcinoma (RCC), bladder cancer, gastric adenocarcinoma, and osteosarcoma. The expression patterns of ZDHHC have been correlated with RCC prognosis, as well as with the immune profiles, molecular features, and signaling pathways of RCC [97]. In bladder cancer, increased palmitoylation levels of FASN and PD‐L1 were correlated with cisplatin resistance [98]. However, it remains to be investigated whether a reduction in the palmitoylation levels of FASN and PD‐L1 can sensitize bladder cancer cells to cisplatin. Besides, in the healthy bladder, ZDHHC2 has been reported to palmitoylate CKAP4 at Cys100, thereby inhibiting epithelial cell proliferation [99] (Fig. 2, Table 1).

Reduced ZDHHC2 expression has been linked to lymph node metastasis and is an independent predictor of poor prognosis in gastric adenocarcinoma [100] (Table 1). In pituitary tumors, E2 treatment promoted ZDHHC7‐ and ZDHHC22‐mediated palmitoylation of ERα at Cys477, enhanced plasma membrane ERα pools, and thus activated ERK1/2, thereby promoting tumor cell proliferation [101, 102, 103] (Fig. 2, Table 1). Taken together, all the above studies indicate that protein palmitoylation is deeply involved in regulating tumorigenesis in a wide range of varied cancer types.

## 12 Shared palmitoylation pattern across cancer types

By summarizing palmitoylated proteins in different types of cancer (Fig. 2 and Table 1), it was found that some key molecules play similar roles in varied types of cancer (Table 2), suggestive of palmitoylation hotspots and possible drug targets for translational application. For instance, EGFR palmitoylation enhances cancer cell migration in breast cancer [32], prostate cancer [93], and NSCLC [83, 84, 85]; KAI1/CD82 palmitoylation prevents angiogenesis and growth in breast cancer [16], inhibits cancer cell migration and invasion in prostate cancer [41], and diminishes bone marrow homing of AML [65]; CD44 palmitoylation suppresses cancer cell migration and metastasis in breast cancer [25] and HCC [96]; KRAS4A palmitoylation facilitates the progression of hematological cancer [59] and pancreatic cancer [80]; NRAS palmitoylation drives cancer progression in hematological cancer [57] and melanoma [75]; PD‐L1 palmitoylation suppresses antitumor immunity and promotes tumor growth in breast cancer [29, 30] and colorectal cancer [17]; and CKAP4 palmitoylation blocks cell proliferation in pancreatic cancer [79] and bladder cancer [99], etc.

**Table 2:** Common palmitoylated proteins in different cancer types.

Common palmitoylated proteins	Type of cancers	Key tumorigenic process
EGFR	Breast cancer, prostate cancer, non‐small cell lung cancer	Apoptosis and migration
CD82	Breast cancer, prostate cancer and hematological cancer	Angiogenesis, metastasis and proliferation
CD44	Breast cancer and hepatocellular carcinoma	Metastasis
KRAS4A	Hematological cancer and pancreatic cancer	Proliferation
NRAS	Hematological cancer and melanoma	Proliferation
PD‐L1	Breast cancer and colorectal cancer	Immunosuppression
CKAP4	Pancreatic cancer and bladder cancer	Proliferation

## 13 Targeting protein palmitoylation in cancer

Since its discovery more than 40 years ago, scientists have gained a more detailed understanding of the biological role and regulation of protein palmitoylation. Abnormal palmitoylation status has been reported in several disease states, such as in neurodegenerative diseases [104, 105], inflammation [106, 107], bacterial and viral infection [108, 109], and human cancers, as described in this review that the involvement of palmitoylation in the progression of almost all cancer types is reflected through the aberrant expression patterns of ZDHHCs and PPTs and through the changes in palmitoylation levels of cancer‐associated proteins, which in turn affect their functions in tumor cell proliferation [39], adhesion [110], migration [111], metastasis [112], and apoptosis [113]. These findings raise the possibility that targeting of either ZDHHCs and PPTs or of palmitoylated cancer‐associated proteins might benefit the treatment of varied cancer types, as discussed above.

### 13.1 Pan‐cancer strategies

For targeting ZDHHCs or PPTs, shRNA or enzyme activity inhibitors were commonly used to either downregulate the protein expression or inhibit the enzymes functioning. For example, inhibiting PPT1 with DC661 also dramatically suppresses the development of a variety of tumors [114]. For targeting a specific palmitoylated protein, palmitoylation‐competitive peptide or species (either small molecule or peptide) to block the specific palmitoylation site was introduced.

### 13.2 Breast cancer

ZDHHC5 regulated Flotillin2 palmitoylation is important for Flotillin2 localization in lipid rafts [115], which serve critical roles in carcinogenesis [116]. Furthermore, it showed that the liver X receptor (LXR) agonist T0901317 disrupted lipid rafts in breast cancer cells by downregulating Flotillin 2 and its membrane‐associated palmitoylation enzyme, ZDHHC5, and the disruption of lipid rafts is linked to the antiproliferative effect of T0901317 [117]. Other examples raised the possibility to target the palmitoylation/depalmitoylation enzymes directly. ZDHHC3 is significantly elevated in both malignant and metastatic human breast cancer, and knockdown of ZDHHC3 with shRNA in MDA‐MB‐231 cells leads to oxidative stress and senescence, enhancing the recruitment of antitumor macrophages and natural killer cells associated with the clearance of senescent tumor cells, thereby reducing xenograft growth in both primary tumors and metastatic lung colonies [118]. Moreover, ZDHHC3 ablation with shRNA in combination with the anticancer drug, poly ADP‐ribose polymerase (PARP) inhibitor PJ‐34, increased oxidative stress and inhibited MDA‐MB‐231 cell proliferation [119]. Similarly, knockdown of depalmitoylating enzyme APT1 (also known as LYPLA1) with shRNA in MDA‐MB‐231 cells depleted a specific subpopulation of tumorigenic cells, resulting in colony formation being suppressed [120], which may be related to the increased apoptosis of cancer cells mediated by CD95 (Fas/APO‐1) [121] (Table 1). Although both ZDHHC3 and APT1 depletion seem to have a similar effect on preventing the proliferation of the same breast cancer cell (MDA‐MB‐231), the molecular mechanisms might be different, as ZDHHC3 regulates oxidative stress and senescence, while APT1 is involved in controlling apoptosis. Further understanding the underlying cause might rely on the identification of their substrates regarding that the opposite roles of ZDHHC3 and APT1 played in protein palmitoylation. Snail is a prominent inducer of epithelial to mesenchymal transition (EMT) and cancer progression [122]. Overexpression of Snail in MCF10A cells induces the expression level of APT enzymes and accelerates depalmitoylation, thereby affecting the palmitoylation cycle of numerous proteins, which may affect breast cancer cell polarity, EMT, and tumor suppression [123]. Taken together, these findings proved in principal that ZDHHC3 and APT1 are potential pharmaceutical targets for treating breast cancer.

### 13.3 Prostate cancer

ZDHHC3‐mediated palmitoylation plays a key role in regulating the expression, stability, and function of integrin α6β4, and knockdown of ZDHHC3 with shRNA affects integrin‐dependent cable formation and signaling in prostate tumor cells [124]. Moreover, ZDHHC14, as a tumor suppressor, is frequently downregulated in testicular germ cell tumors and prostate cancer, and overexpression of ZDHHC14 resulted in inhibited cell viability and promoted cell apoptosis [125] (Table 1). Last, but not least, blocking EGFR palmitoylation (mutation of Cys797, Cys1025, and Cys1122 to alanine) disrupted TKI‐induced EGFR dimerization, resulting in the apoptosis of TKI‐resistant cancer cells [93].

### 13.4 Colorectal cancer

Cancer stem cells (CSCs) are types of tumor cells with self‐renewal and multi‐differentiation capabilities, which play a key role in tumor progression. One study illustrated that a small‐molecule inhibitor, RU‐SKI 43, targets SHH‐palmitoylating Hedgehog acyltransferase (HHAT), and effectively reduces colon CSCs survival at low dosages by blocking all signaling downstream of SHH [126]. Surprisingly, another group showed that dual MEK‐PI3K inhibitor therapy somehow significantly reduced KRAS mutated mucin 2 (MUC2) expression, MUC2 palmitoylation, and secretion in colon cells, ultimately suppressing mucinous tumor growth *in vivo* [127]. Last, but briefly, the overexpression of ZDHHC9 could dramatically suppress the proliferation of colorectal cancer cells [128] (Table 1). However, it should be noted that more evidence from xenograft mouse models and clinical verifications are still needed to validate these potentially important findings.

### 13.5 Hematological cancers

It was revealed that the universal palmitoylation inhibitor 2‐Bromopalmitate (2‐BP) and all‐trans retinoic acid have a synergistic differentiation‐inducing effect in acute promyelocytic leukemia. Mechanically, it was suggested that 2‐BP covalently binds to retinoic acid receptor alpha (RARα) and prevents RARα degradation, resulting in the promoted transcription of RARα‐target genes [129]. This suggests that 2‐BP has potential in treating leukemia, although it should be noted that 2‐BP lacks substrate specificity, and therefore might bring unexpected toxicity *in vivo*. In addition, the combination of the nonspecific depalmitoylation inhibitor palmostatin B (Palm‐B) with gilteritinib (FLT3 inhibitor) also significantly suppressed FLT3‐ITD‐mediated signaling and leukemia progression [60], indicating that raising the level of palmitoylation might also bring beneficial effects in leukemia. On the side of PPTs, CD95, a member of the tumor necrosis factor superfamily, has been demonstrated to bind to its ligand (CD95L) and transmit a death signal to cells, causing apoptosis [130]. However, tumor cells escape CD95‐mediated apoptosis by silencing CD95 expression [131]. Therefore, knockdown of APT1 and APT2 with siRNA or pharmacological inhibition of APT1 and APT2 using Palm‐B, or overexpressing miRs‐138/‐424 to deregulate and target APT1 and APT2, restores CD95‐mediated apoptosis in chronic lymphocytic leukemia cells [121]. Additionally, ABD957, a strong and selective covalent inhibitor of the ABHD17A/B/C, inhibits NRAS depalmitoylation in AML cells, resulting in suppressed NRAS signaling and the development of NRAS‐mutant AML [132] (Table 1). These examples demonstrated that the regulatory roles of palmitoylation in hematological cancers can be bidirectional, and hence the targeting strategies should be designed carefully case‐by‐case.

### 13.6 Melanoma

It was reported that inhibition of PPT1 with specific PPT1 inhibitors hydroxychloroquine (HCQ) or dimeric CQ DC661 induced interferon‐β secretion in macrophages and enhanced the antitumor efficacy of the anti‐PD‐1 antibody in melanoma [133]. In addition, a mono‐palmitoylated peptide that contained the dominant MHC class‐I and II epitopes of the human melanoma antigen gp100 (gp100^280‐288/45‐59^) or the mouse model antigen ovalbumin (OVA^257‐264/323‐339^) conjugated to palmitolate anhydride (C16:0) was developed that is efficiently internalized by DCs and subsequently cross‐presented to CD8^+^ T‐cells, ultimately inhibiting melanoma progression [134] (Table 1).

### 13.7 Pancreatic cancer

A potent antitumor compound, DQ661, has been identified, which specifically targets and inhibits PPT1 in lysosomes, resulting in rapid accumulation of palmitoylated proteins, thereby impairing mTOR and lysosomal catabolism, which in turn significantly inhibiting tumor growth in mouse models of melanoma, colorectal, and pancreatic cancer [135] (Table 1).

### 13.8 Lung cancer

A study illustrated that knockdown of ZDHHC5 dramatically inhibited cell proliferation, colony formation and cell invasion *in vitro*, as well as severely hindered NSCLC tumor xenograft formation, demonstrating the oncogenic capacity of ZDHHC5 [136]. Similarly, another study found that silencing APT1 with shRNA significantly reduced NSCLC cell proliferation, migration, and invasion *in vitro* [137] (Table 1). Disclosing the enzymatic substrates of ZDHHC5 and APT1 will be the key to understand why they play similar roles in lung cancer.

### 13.9 Glioblastoma

Similar to protein palmitoylation inhibitors, substrate‐analog inhibitors (similar in structure to one of the substrates of the reaction they are inhibiting, e.g. 2‐BP, cerulenin or tunicamycin) significantly suppressed GBM cell survival by inhibiting the cell cycle and promoting apoptosis [138] (Table 1). Interestingly, a study showed that local anesthetics could attenuate glioblastoma stem cell proliferation and self‐renewal via diminishing ZDHHC15‐mediated palmitoylation of interleukin 6 signal transducer (IL6ST, also named GP130) [139]. Yet, the molecular connections between local anesthetics and ZDHHC15 activity remain to be further examined.

### 13.10 Ovarian cancer

Two selective and potent FASN inhibitors, TVB‐3166 and TVB‐3664, were discovered and used in ovarian, lung, prostate and pancreatic cancer mouse models, which presented that inhibiting FASN in combination with taxane therapy inhibited tumor cell growth both *in vitro* and *in vivo*, possibly by disrupting tubulin palmitoylation, expression and microtubule organization [140]. Mechanistically, it is negotiable how exactly TVB‐3166 exerts its antitumor effect as another study showed that TVB‐3166 treatment alters ERα subcellular localization and reduces ERα levels by inducing endoplasmic reticulum stress, and exhibits antitumor activity in tamoxifen‐resistant breast tumor cells [141] (Table 1). Further investigations are warranted on this issue.

### 13.11 Other cancer types

For the treatment of HCC, GNS561, a new lysosomotropic small molecule drug was developed to target PPT1 to modulate lysosomal deacidification and inhibit mammalian target of rapamycin (mTOR) signaling pathway. Moreover, GNS561 and sorafenib (a multikinase inhibitor) were found to have a synergistic impact on preventing tumor development and cell proliferation in a mouse tumor model [142, 143] (Table 1). In addition, the combination of adriamycin (an antibiotic drug) and 2‐BP strongly inhibited the proliferation of osteosarcoma cell lines and primary osteosarcoma cells [144] (Table 1).

## 14 Future perspectives

First, the specific substrates for ZDHHCs and PPTs have not yet been fully identified. So far, the substrates of APT1 and PPT1 have been identified in the human embryonic kidney cell line HEK293T [145] and in the human neuroblastoma cell line SH‐SY5Y [146], while some substrates of other ZDHHCs and PPTs have been reported [13, 14, 15, 69, 90, 115, 132, 139, 147, 148]. Several studies using palm‐proteomics (isolation of total palmitoylated proteins + mass spectrometry) have successfully identified a spectrum of substrates for ZDHHC3 in breast and prostate cancer cells [119], indicating that ZDHHCs and PPTs have multiple substrates, which also vary depending on cancer type. Moreover, in our experience, palm‐proteomics analyses may give false‐positive results, and further validation by Acyl‐Biotin Exchange (ABE)/Acyl‐RAC and point‐mutagenesis are necessary. Interestingly, ZDHHC family members appear to differ from the protein structure perspective, and these differences may confer specificity in substrates recognition. For example, ZDHHC5 and ZDHHC8 possess a long and highly disordered C‐terminal tail that can interact with structural aspects of many proteins, such as the PDZ domain; whereas ZDHHC17 and ZDHHC13 contain an ankyrin repeat domain, which enables them to interact with many known ZDHHC17 substrates, including huntingtin, SNAP25, and cysteine chain proteins [149]. Understanding this from such an angle might facilitate clarifying the substrates complexity of ZDHHCs/PPTs, although much effort is needed.

Second, only a few selective inhibitors targeting ZDHHCs, PPTs, or palmitoylated proteins have been developed to date. As palmitoylation may regulate tumorigenesis in both directions (promoting or inhibiting cancer progression), palmitoylation‐targeting strategies should be carefully designed according to each specific situation.

Initially, several studies reported that 2‐BP was used to inhibit tumorigenesis at a cellular level [150]; however, the inhibition of protein lipidation is toxic to normal cells, which might limit the application of 2‐BP in cancer patients. Furthermore, as both oncogenes and tumor suppressors can be palmitoylated, the application of 2‐BP might compromise its treatment effect due to the lack of selectivity. Later on, Palm‐B was developed to selectively target APT1, but eventually it was found to target APT2 as well. To specifically inhibit each of the two APTs, the selective APT1 and APT2 inhibitors ML348 and ML349 were developed. Recently, the ABHD17A/B/C inhibitor ABD957 as well as the PPT1 inhibitors DC661, DQ661, and GNS561 were also identified [69, 114, 132, 133, 135, 142, 143, 151]. Yet the target specificities of these “newly” identified inhibitors need to be carefully validated.

No specific and potent inhibitors have so far been developed to target ZDHHCs. However, considering that ZDHHCs might have multiple and even cross‐substrates for palmitoylation [149], potential ZDHHCs inhibitors might face an unexpected outcome, and thus possibly reduce their translational values in clinics [6]. Alternatively, other strategies, such as developing small‐molecule inhibitors targeting palmitoylation insertions and AMD have been reported [23, 75]. Indeed, a competitive inhibitor against specific sites of palmitoylation showed a good inhibitory efficacy in colorectal cancer [17]; additionally, an interesting vaccination strategy using mono‐palmitic acid modified antigenic peptide that could significantly facilitate antitumor immunity in melanoma [134]. However, more successful examples are awaited to prove in principal that these strategies are truly effective both *in vitro* and *in vivo*.

Third, it is difficult to establish a direct mechanistic connection between palmitoylation of specific proteins and their function in cancer. This stems from the lack of specific palmitoyl transferase inhibitors, of palmitoyl‐mimetic mutations, or of consensus palmitoylation sequences that make gain of function studies challenging. Moreover, there are no antibodies to palmitoylated epitopes, and therefore correlation between palmitoylation levels of a protein and tumor grade in patient samples are extremely difficult. It should be noted that most of the examples summarized here are *in vitro* studies in cell lines. Therefore, introducing a palmitoylation antibody and novel tools as specific agonist or antagonist of palmitoylation will greatly advance the research fields.

Together, it can be visualized that the functional studies of palmitoylation in tumorigenesis are mostly focused on breast cancer, prostate cancer, colorectal cancer, leukemia, melanoma, pancreatic cancer, and NSCLC, but are less explored in other types of cancer, including RCC, osteosarcoma, GBM, ovarian cancer, HCC, bladder carcinoma, gastric adenocarcinoma, and pituitary tumors. More pathogenic mechanisms related to protein palmitoylation are expected to be discovered in all these human cancer types, which will give a solid theoretical basis for therapeutic development in the future.

## Conflict of interest

The authors declare no conflicts of interest.

## Author Contributions

BZ, QH, and EK conceived and designed the project, BZ and EK wrote the article, and BZ, YL, and EK revised the article.

## References

[1] Hanahan D . Hallmarks of cancer: new dimensions. Cancer Discov. 2022;12:31–46. 35022204 10.1158/2159-8290.CD-21-1059

[2] Lothrop AP , Torres MP , Fuchs SM . Deciphering post‐translational modification codes. FEBS Lett. 2013;587:1247–57. 23402885 10.1016/j.febslet.2013.01.047 PMC3888991

[3] Fhu CW , Ali A . Protein lipidation by palmitoylation and myristoylation in cancer. Front Cell Dev Biol. 2021;9:673647. 34095144 10.3389/fcell.2021.673647 PMC8173174

[4] Linder ME , Deschenes RJ . Palmitoylation: policing protein stability and traffic. Nat Rev Mol Cell Biol. 2007;8:74–84. 17183362 10.1038/nrm2084

[5] Kim YC , Lee SE , Kim SK , Jang HD , Hwang I , Jin S , et al. Toll‐like receptor mediated inflammation requires FASN‐dependent MYD88 palmitoylation. Nat Chem Biol. 2019;15:907–16. 31427815 10.1038/s41589-019-0344-0

[6] Ko PJ , Dixon SJ . Protein palmitoylation and cancer. EMBO Rep. 2018;19:e46666. 30232163 10.15252/embr.201846666 PMC6172454

[7] Baekkeskov S , Kanaani J . Palmitoylation cycles and regulation of protein function. Mol Membr Biol. 2009;26:42–54. 19169934 10.1080/09687680802680108

[8] Kaur I , Yarov‐Yarovoy V , Kirk LM , Plambeck KE , Barragan EV , Ontiveros ES , et al. Activity‐dependent palmitoylation controls synDIG1 stability, localization, and function. J Neurosci. 2016;36:7562–8. 27445135 10.1523/JNEUROSCI.4859-14.2016 PMC4951570

[9] Jiang H , Zhang X , Chen X , Aramsangtienchai P , Tong Z , Lin H . Protein lipidation: occurrence, mechanisms, biological functions, and enabling technologies. Chem Rev. 2018;118:919–88. 29292991 10.1021/acs.chemrev.6b00750 PMC5985209

[10] Zhu YC , Li D , Wang L , Lu B , Zheng J , Zhao SL , et al. Palmitoylation‐dependent CDKL5‐PSD‐95 interaction regulates synaptic targeting of CDKL5 and dendritic spine development. Proc Natl Acad Sci USA. 2013;110:9118–23. 23671101 10.1073/pnas.1300003110 PMC3670390

[11] Greaves J , Chamberlain LH . Palmitoylation‐dependent protein sorting. J Cell Biol. 2007;176:249–54. 17242068 10.1083/jcb.200610151 PMC2063950

[12] Thuma F , Heiler S , Schnölzer M , Zöller M . Palmitoylated claudin7 captured in glycolipid‐enriched membrane microdomains promotes metastasis via associated transmembrane and cytosolic molecules. Oncotarget. 2016;7:30659–77. 27120791 10.18632/oncotarget.8928 PMC5058708

[13] Zhang ZX , Li X , Yang F , Chen C , Liu P , Ren Y , et al. DHHC9‐mediated GLUT1 S‐palmitoylation promotes glioblastoma glycolysis and tumorigenesis. Nat Commun. 2021;12:5872. 34620861 10.1038/s41467-021-26180-4 PMC8497546

[14] Pei X , Li KY , Shen Y , Li JT , Lei MZ , Fang CY , et al. Palmitoylation of MDH2 by ZDHHC18 activates mitochondrial respiration and accelerates ovarian cancer growth. Sci China Life Sci. 2022 [Online ahead of print]. 10.1007/s11427-021-2048-2 35366151

[15] Chen S , Zhu B , Yin C , Liu W , Han C , Chen B , et al. Palmitoylation‐dependent activation of MC1R prevents melanomagenesis. Nature. 2017;549:399–403. 28869973 10.1038/nature23887 PMC5902815

[16] Lee JW , Hur J , Kwon YW , Chae CW , Choi JI , Hwang I , et al. KAI1(CD82) is a key molecule to control angiogenesis and switch angiogenic milieu to quiescent state. J Hematol Oncol. 2021;14:148. 34530889 10.1186/s13045-021-01147-6 PMC8444549

[17] Yao H , Lan J , Li CS , Shi HB , Brosseau JP , Wang HB , et al. Inhibiting PD‐L1 palmitoylation enhances T‐cell immune responses against tumours. Nat Biomed Eng. 2019;3:306–17. 30952982 10.1038/s41551-019-0375-6

[18] Yeste‐Velasco M , Linder ME , Lu YJ . Protein S‐palmitoylation and cancer. Biochim Biophys Acta. 2015;1856:107–20. 26112306 10.1016/j.bbcan.2015.06.004

[19] Resh MD . Palmitoylation of proteins in cancer. Biochem Soc Trans. 2017;45:409–16. 28408481 10.1042/BST20160233

[20] Liu Z , Xiao M , Mo Y , Wang H , Han Y , Zhao X , et al. Emerging roles of protein palmitoylation and its modifying enzymes in cancer cell signal transduction and cancer therapy. Int J Biol Sci. 2022;18:3447–57. 35637973 10.7150/ijbs.72244 PMC9134921

[21] Barylko B , Mao YS , Wlodarski P , Jung G , Binns DD , Sun HQ , et al. Palmitoylation controls the catalytic activity and subcellular distribution of phosphatidylinositol 4‐kinase II{alpha}. J Biol Chem. 2009;284:9994–10003. 19211550 10.1074/jbc.M900724200 PMC2665123

[22] Li J , Lu Y , Zhang J , Kang H , Qin Z , Chen C . PI4KIIα is a novel regulator of tumor growth by its action on angiogenesis and HIF‐1α regulation. Oncogene. 2010;29:2550–9. 20154717 10.1038/onc.2010.14

[23] Li J , Gao Z , Zhao D , Zhang L , Qiao X , Zhao Y , et al. PI‐273, a substrate‐competitive, specific small‐molecule inhibitor of PI4KIIalpha, inhibits the growth of breast cancer cells. Cancer Res. 2017;77:6253–66. 28827373 10.1158/0008-5472.CAN-17-0484

[24] Heakal Y , Woll MP , Fox T , Seaton K , Levenson R , Kester M . Neurotensin receptor‐1 inducible palmitoylation is required for efficient receptor‐mediated mitogenic‐signaling within structured membrane microdomains. Cancer Biol Ther. 2011;12:427–35. 21725197 10.4161/cbt.12.5.15984 PMC3219081

[25] Babina IS , McSherry EA , Donatello S , Hill ADK , Hopkins AM . A novel mechanism of regulating breast cancer cell migration via palmitoylation‐dependent alterations in the lipid raft affiliation of CD44. Breast Cancer Res. 2014;16:R19. 24512624 10.1186/bcr3614 PMC3978828

[26] Coleman DT , Soung YH , Surh YJ , Cardelli JA , Chung J . Curcumin prevents palmitoylation of integrin beta4 in breast cancer cells. PLoS One. 2015;10:e0125399. 25938910 10.1371/journal.pone.0125399 PMC4418632

[27] Yan W , Huang J , Zhang Q , Zhang J . Role of metastasis suppressor KAI1/CD82 in different cancers. J Oncol. 2021;2021:9924473. 34306081 10.1155/2021/9924473 PMC8285166

[28] Christgen M , Bruchhardt H , Ballmaier M , Krech T , Langer F , Kreipe H , et al. KAI1/CD82 is a novel target of estrogen receptor‐mediated gene repression and downregulated in primary human breast cancer. Int J Cancer. 2008;123:2239–46. 18712725 10.1002/ijc.23806

[29] Zou W , Wolchok JD , Chen L . PD‐L1 (B7‐H1) and PD‐1 pathway blockade for cancer therapy: mechanisms, response biomarkers, and combinations. Sci Transl Med. 2016;8:328rv4. 10.1126/scitranslmed.aad7118 PMC4859220 26936508

[30] Yang Y , Hsu JM , Sun LL , Chan LC , Li CW , Hsu JL , et al. Palmitoylation stabilizes PD‐L1 to promote breast tumor growth. Cell Res. 2019;29:83–6. 30514902 10.1038/s41422-018-0124-5 PMC6318320

[31] Menendez JA , Lupu R . Fatty acid synthase and the lipogenic phenotype in cancer pathogenesis. Nat Rev Cancer. 2007;7:763–77. 17882277 10.1038/nrc2222

[32] Bollu LR , Ren J , Blessing AM , Katreddy RR , Gao G , Xu L , et al. Involvement of de novo synthesized palmitate and mitochondrial EGFR in EGF induced mitochondrial fusion of cancer cells. Cell Cycle. 2014;13:2415–30. 25483192 10.4161/cc.29338 PMC4128886

[33] Martin GS . The hunting of the Src. Nat Rev Mol Cell Biol. 2001;2:467–75. 11389470 10.1038/35073094

[34] Irby RB , Yeatman TJ . Role of Src expression and activation in human cancer. Oncogene. 2000;19:5636–42. 11114744 10.1038/sj.onc.1203912

[35] Cai H , Smith DA , Memarzadeh S , Lowell CA , Cooper JA , Witte ON . Differential transformation capacity of Src family kinases during the initiation of prostate cancer. Proc Natl Acad Sci USA. 2011;108:6579–84. 21464326 10.1073/pnas.1103904108 PMC3080985

[36] Fujita K , Nonomura N . Role of androgen receptor in prostate cancer: a review. World J Mens Health. 2019;37:288–95. 30209899 10.5534/wjmh.180040 PMC6704300

[37] Aurilio G , Cimadamore A , Mazzucchelli R , Lopez‐Beltran A , Verri E , Scarpelli M , et al. Androgen receptor signaling pathway in prostate cancer: from genetics to clinical applications. Cell. 2020;9:2653. 10.3390/cells9122653 PMC7763510 33321757

[38] Yang X , Guo Z , Sun F , Li W , Alfano A , Shimelis H , et al. Novel membrane‐associated androgen receptor splice variant potentiates proliferative and survival responses in prostate cancer cells. J Biol Chem. 2011;286:36152–60. 21878636 10.1074/jbc.M111.265124 PMC3195613

[39] Cui L , Liu M , Lai S , Hou H , Diao T , Zhang D , et al. Androgen upregulates the palmitoylation of eIF3L in human prostate LNCaP cells. Onco Targets Ther. 2019;12:4451–9. 31239713 10.2147/OTT.S193480 PMC6556480

[40] Li W , Zhang J , Zou L , Cui J , Su F , Jin J , et al. Palmitoylome profiling indicates that androgens regulate the palmitoylation of alphatubulin in prostate cancerderived LNCaP cells and supernatants. Oncol Rep. 2019;42:2788–96. 31578588 10.3892/or.2019.7333

[41] Zhou B , Liu L , Reddivari M , Zhang XA . The palmitoylation of metastasis suppressor KAI1/CD82 is important for its motility‐ and invasiveness‐inhibitory activity. Cancer Res. 2004;64:7455–63. 15492270 10.1158/0008-5472.CAN-04-1574

[42] Fiorentino M , Zadra G , Palescandolo E , Fedele G , Bailey D , Fiore C , et al. Overexpression of fatty acid synthase is associated with palmitoylation of Wnt1 and cytoplasmic stabilization of beta‐catenin in prostate cancer. Lab Invest. 2008;88:1340–8. 18838960 10.1038/labinvest.2008.97 PMC3223737

[43] Di Vizio D , Adam RM , Kim J , Kim R , Sotgia F , Williams T , et al. Caveolin‐1 interacts with a lipid raft‐associated population of fatty acid synthase. Cell Cycle. 2008;7:2257–67. 18635971 10.4161/cc.7.14.6475

[44] De Piano M , Manuelli V , Zadra G , Otte J , Edqvist PD , Ponten F , et al. Lipogenic signalling modulates prostate cancer cell adhesion and migration via modification of Rho GTPases. Oncogene. 2020;39:3666–79. 32139877 10.1038/s41388-020-1243-2 PMC7190568

[45] Galluzzo P , Caiazza F , Moreno S , Marino M . Role of ERbeta palmitoylation in the inhibition of human colon cancer cell proliferation. Endocr Relat Cancer. 2007;14:153–67. 17395984 10.1677/ERC-06-0020

[46] Caiazza F , Galluzzo P , Lorenzetti S , Marino M . 17Beta‐estradiol induces ERbeta up‐regulation via p38/MAPK activation in colon cancer cells. Biochem Biophys Res Commun. 2007;359:102–7. 17524358 10.1016/j.bbrc.2007.05.059

[47] Greenlee JD , Lopez‐Cavestany M , Ortiz‐Otero N , Liu K , Subramanian T , Cagir B , et al. Oxaliplatin resistance in colorectal cancer enhances TRAIL sensitivity via death receptor 4 upregulation and lipid raft localization. Elife. 2021;10:e67750. 34342264 10.7554/eLife.67750 PMC8331188

[48] von Karstedt S , Montinaro A , Walczak H . Exploring the TRAILs less travelled: TRAIL in cancer biology and therapy. Nat Rev Cancer. 2017;17:352–66. 28536452 10.1038/nrc.2017.28

[49] Rossin A , Durivault J , Chakhtoura‐Feghali T , Lounnas N , Gagnoux‐Palacios L , Hueber AO . Fas palmitoylation by the palmitoyl acyltransferase DHHC7 regulates Fas stability. Cell Death Differ. 2015;22:643–53. 25301068 10.1038/cdd.2014.153 PMC4356335

[50] Taciak B , Pruszynska I , Kiraga L , Bialasek M , Krol M . Wnt signaling pathway in development and cancer. J Physiol Pharmacol. 2018;69:185–96. 10.26402/jpp.2018.2.07 29980141

[51] Klaus C , Schneider U , Hedberg C , Schutz AK , Bernhagen J , Waldmann H , et al. Modulating effects of acyl‐CoA synthetase 5‐derived mitochondrial Wnt2B palmitoylation on intestinal Wnt activity. World J Gastroenterol. 2014;20:14855–64. 25356045 10.3748/wjg.v20.i40.14855 PMC4209548

[52] Dubois F , Leroy C , Simon V , Benistant C , Roche S . YES oncogenic activity is specified by its SH4 domain and regulates RAS‐MAPK signaling in colon carcinoma cells. Am J Cancer Res. 2015;5:1972–87. 26269757 PMC4529617

[53] Kyuno D , Zhao K , Schnolzer M , Provaznik J , Hackert T , Zoller M . Claudin7‐dependent exosome‐promoted reprogramming of nonmetastasizing tumor cells. Int J Cancer. 2019;145:2182–200. 30945750 10.1002/ijc.32312

[54] Du W , Hua F , Li X , Zhang J , Li S , Wang W , et al. Loss of optineurin drives cancer immune evasion via palmitoylation‐dependent IFNGR1 lysosomal sorting and degradation. Cancer Discov. 2021;11:1826–43. 33627378 10.1158/2159-8290.CD-20-1571 PMC8292167

[55] Fernandez‐Medarde A , Santos E . Ras in cancer and developmental diseases. Genes Cancer. 2011;2:344–58. 21779504 10.1177/1947601911411084 PMC3128640

[56] Li H , Yu X , Liu X , Hu P , Shen L , Zhou Y , et al. Wogonoside induces depalmitoylation and translocation of PLSCR1 and N‐RAS in primary acute myeloid leukaemia cells. J Cell Mol Med. 2018;22:2117–30. 29377576 10.1111/jcmm.13481 PMC5867108

[57] Cuiffo B , Ren R . Palmitoylation of oncogenic NRAS is essential for leukemogenesis. Blood. 2010;115:3598–605. 20200357 10.1182/blood-2009-03-213876 PMC2867268

[58] Laude AJ , Prior IA . Palmitoylation and localisation of RAS isoforms are modulated by the hypervariable linker domain. J Cell Sci. 2008;121:421–7. 18211960 10.1242/jcs.020107

[59] Zhao H , Liu P , Zhang R , Wu M , Li D , Zhao X , et al. Roles of palmitoylation and the KIKK membrane‐targeting motif in leukemogenesis by oncogenic KRAS4A. J Hematol Oncol. 2015;8:132. 26715448 10.1186/s13045-015-0226-1 PMC4696201

[60] Lv K , Ren JG , Han X , Gui J , Gong C , Tong W . Depalmitoylation rewires FLT3‐ITD signaling and exacerbates leukemia progression. Blood. 2021;138:2244–55. 34111291 10.1182/blood.2021011582 PMC8832469

[61] dos Santos GA , Thome CH , Ferreira GA , Yoneda JS , Nobre TM , Daghastanli KR , et al. Interaction of 10‐(octyloxy) decyl‐2‐(trimethylammonium) ethyl phosphate with mimetic membranes and cytotoxic effect on leukemic cells. Biochim Biophys Acta. 2010;1798:1714–23. 20488162 10.1016/j.bbamem.2010.05.013

[62] Thome CH , dos Santos GA , Ferreira GA , Scheucher PS , Izumi C , Leopoldino AM , et al. Linker for activation of T‐cell family member2 (LAT2) a lipid raft adaptor protein for AKT signaling, is an early mediator of alkylphospholipid anti‐leukemic activity. Mol Cell Proteomics. 2012;11:1898–912. 23001822 10.1074/mcp.M112.019661 PMC3518132

[63] Guo F , Luo Y , Jiang X , Lu X , Roberti D , Lossos C , et al. Recent BCR stimulation induces a negative autoregulatory loop via FBXO10 mediated degradation of HGAL. Leukemia. 2020;34:553–66. 31570756 10.1038/s41375-019-0579-5

[64] Termini CM , Lidke KA , Gillette JM . Tetraspanin CD82 regulates the spatiotemporal dynamics of PKCalpha in acute myeloid leukemia. Sci Rep. 2016;6:29859. 27417454 10.1038/srep29859 PMC4945921

[65] Marjon KD , Termini CM , Karlen KL , Saito‐Reis C , Soria CE , Lidke KA , et al. Tetraspanin CD82 regulates bone marrow homing of acute myeloid leukemia by modulating the molecular organization of N‐cadherin. Oncogene. 2016;35:4132–40. 26592446 10.1038/onc.2015.449 PMC4877306

[66] Tohumeken S , Baur R , Bottcher M , Stoll A , Loschinski R , Panagiotidis K , et al. Palmitoylated proteins on AML‐derived extracellular vesicles promote myeloid‐derived suppressor cell differentiation via TLR2/Akt/mTOR signaling. Cancer Res. 2020;80:3663–76. 32605996 10.1158/0008-5472.CAN-20-0024

[67] Wolf Horrell EM , Boulanger MC , D'Orazio JA . Melanocortin 1 receptor: structure, function, and regulation. Front Genet. 2016;7:95. 27303435 10.3389/fgene.2016.00095 PMC4885833

[68] Raimondi S , Sera F , Gandini S , Iodice S , Caini S , Maisonneuve P , et al. MC1R variants, melanoma and red hair color phenotype: a meta‐analysis. Int J Cancer. 2008;122:2753–60. 18366057 10.1002/ijc.23396

[69] Chen S , Han C , Miao X , Li X , Yin C , Zou J , et al. Targeting MC1R depalmitoylation to prevent melanomagenesis in redheads. Nat Commun. 2019;10:877. 30787281 10.1038/s41467-019-08691-3 PMC6382811

[70] Sadeghi RS , Kulej K , Kathayat RS , Garcia BA , Dickinson BC , Brady DC , et al. Wnt5a signaling induced phosphorylation increases APT1 activity and promotes melanoma metastatic behavior. Elife. 2018;7:e34362. 29648538 10.7554/eLife.34362 PMC5919757

[71] Wang W , Runkle KB , Terkowski SM , Ekaireb RI , Witze ES . Protein depalmitoylation is induced by Wnt5a and promotes polarized cell behavior. J Biol Chem. 2015;290:15707–16. 25944911 10.1074/jbc.M115.639609 PMC4505481

[72] Rodenhuis S . Ras and human tumors. Semin Cancer Biol. 1992;3:241–7. 1421168

[73] Billadeau D , Liu P , Jelinek D , Shah N , LeBien TW , Van Ness B . Activating mutations in the N‐ and K‐ras oncogenes differentially affect the growth properties of the IL‐6‐dependent myeloma cell line ANBL6. Cancer Res. 1997;57:2268–75. 9187131

[74] Papke B , Der CJ . Drugging RAS: know the enemy. Nature. 2017;355:1158–63. 10.1126/science.aam7622 28302824

[75] Vora HD , Johnson M , Brea RJ , Rudd AK , Devaraj NK . Inhibition of NRAS signaling in melanoma through direct depalmitoylation using amphiphilic nucleophiles. ACS Chem Biol. 2020;15:2079–86. 32568509 10.1021/acschembio.0c00222 PMC7556697

[76] Sanders SS , Martin DD , Butland SL , Lavallee‐Adam M , Calzolari D , Kay C , et al. Curation of the mammalian palmitoylome indicates a pivotal role for palmitoylation in diseases and disorders of the nervous system and cancers. PLoS Comput Biol. 2015;11:e1004405. 26275289 10.1371/journal.pcbi.1004405 PMC4537140

[77] Kimura H , Yamamoto H , Harada T , Fumoto K , Osugi Y , Sada R , et al. CKAP4, a DKK1 receptor, is a biomarker in exosomes derived from pancreatic cancer and a molecular target for therapy. Clin Cancer Res. 2019;25:1936–47. 30610103 10.1158/1078-0432.CCR-18-2124

[78] Raisch J , Cote‐Biron A , Rivard N . A role for the WNT co‐receptor LRP6 in pathogenesis and therapy of epithelial cancers. Cancers (Basel). 2019;11:1162. 31412666 10.3390/cancers11081162 PMC6721565

[79] Sada R , Kimura H , Fukata Y , Fukata M , Yamamoto H , Kikuchi A . Dynamic palmitoylation controls the microdomain localization of the DKK1 receptors CKAP4 and LRP6. Sci Signal. 2019;12:eaat9519. 31744930 10.1126/scisignal.aat9519

[80] Amendola CR , Mahaffey JP , Parker SJ , Ahearn IM , Chen WC , Zhou M , et al. KRAS4A directly regulates hexokinase 1. Nature. 2019;576:482–6. 31827279 10.1038/s41586-019-1832-9 PMC6923592

[81] Sun C , Wang P , Dong W , Liu H , Sun J , Zhao L . LncRNA PVT1 promotes exosome secretion through YKT6, RAB7, and VAMP3 in pancreatic cancer. Aging (Albany NY). 2020;12:10427–40. 32499447 10.18632/aging.103268 PMC7346024

[82] Owonikoko TK , Ragin CC , Belani CP , Oton AB , Gooding WE , Taioli E , et al. Lung cancer in elderly patients: an analysis of the surveillance, epidemiology, and end results database. J Clin Oncol. 2007;25:5570–7. 18065729 10.1200/JCO.2007.12.5435

[83] Runkle KB , Kharbanda A , Stypulkowski E , Cao XJ , Wang W , Garcia BA , et al. Inhibition of DHHC20‐mediated EGFR palmitoylation creates a dependence on EGFR signaling. Mol Cell. 2016;62:385–96. 27153536 10.1016/j.molcel.2016.04.003 PMC4860254

[84] Kharbanda A , Walter DM , Gudiel AA , Schek N , Feldser DM , Witze ES . Blocking EGFR palmitoylation suppresses PI3K signaling and mutant KRAS lung tumorigenesis. Sci Signal. 2020;13:eaax2364. 32127496 10.1126/scisignal.aax2364 PMC7310254

[85] Ali A , Levantini E , Teo JT , Goggi J , Clohessy JG , Wu CS , et al. Fatty acid synthase mediates EGFR palmitoylation in EGFR mutated non‐small cell lung cancer. EMBO Mol Med. 2018;10:e8313. 29449326 10.15252/emmm.201708313 PMC5840543

[86] Fan X , Fan J , Yang H , Zhao C , Niu W , Fang Z , et al. Heterogeneity of subsets in glioblastoma mediated by Smad3 palmitoylation. Oncogenesis. 2021;10:72. 34707087 10.1038/s41389-021-00361-8 PMC8551152

[87] Tong H , Yu X , Lu X , Wang P . Downregulation of solute carriers of glutamate in gliosomes and synaptosomes may explain local brain metastasis in anaplastic glioblastoma. IUBMB Life. 2015;67:306–11. 25914026 10.1002/iub.1372

[88] Vander Heiden MG , Cantley LC , Thompson CB . Understanding the Warburg effect: the metabolic requirements of cell proliferation. Science. 2009;324:1029–33. 19460998 10.1126/science.1160809 PMC2849637

[89] Thorens B , Mueckler M . Glucose transporters in the 21st Century. Am J Physiol Endocrinol Metab. 2010;298:E141–5. 20009031 10.1152/ajpendo.00712.2009 PMC2822486

[90] Yuan M , Chen X , Sun Y , Jiang L , Xia Z , Ye K , et al. ZDHHC12‐mediated claudin‐3 S‐palmitoylation determines ovarian cancer progression. Acta Pharm Sin B. 2020;10:1426–39. 32963941 10.1016/j.apsb.2020.03.008 PMC7488353

[91] Tavsan Z , Ayar Kayali H . EpCAM‐claudin‐tetraspanin‐modulated ovarian cancer progression and drug resistance. Cell Adh Migr. 2020;14:57–68. 32091301 10.1080/19336918.2020.1732761 PMC7757826

[92] Chen B , Zheng B , DeRan M , Jarugumilli GK , Fu J , Brooks YS , et al. ZDHHC7‐mediated S‐palmitoylation of Scribble regulates cell polarity. Nat Chem Biol. 2016;12:686–93. 27380321 10.1038/nchembio.2119 PMC4990496

[93] Thomas R , Srivastava S , Katreddy RR , Sobieski J , Weihua Z . Kinase‐inactivated EGFR is required for the survival of wild‐type EGFR‐expressing cancer cells treated with tyrosine kinase inhibitors. Int J Mol Sci. 2019;20:2515. 31121829 10.3390/ijms20102515 PMC6566606

[94] Adams MN , Harrington BS , He Y , Davies CM , Wallace SJ , Chetty NP , et al. EGF inhibits constitutive internalization and palmitoylation‐dependent degradation of membrane‐spanning procancer CDCP1 promoting its availability on the cell surface. Oncogene. 2015;34:1375–83. 24681947 10.1038/onc.2014.88

[95] Forner A , Reig M , Bruix J . Hepatocellular carcinoma. Lancet. 2018;391:1301–14. 29307467 10.1016/S0140-6736(18)30010-2

[96] Yang Z , Qin W , Chen Y , Yuan B , Song X , Wang B , et al. Cholesterol inhibits hepatocellular carcinoma invasion and metastasis by promoting CD44 localization in lipid rafts. Cancer Lett. 2018;429:66–77. 29746928 10.1016/j.canlet.2018.04.038

[97] Liu Z , Liu C , Xiao M , Han Y , Zhang S , Xu B . Bioinformatics analysis of the prognostic and biological significance of ZDHHC‐protein acyltransferases in kidney renal clear cell carcinoma. Front Oncol. 2020;10:565414. 33364189 10.3389/fonc.2020.565414 PMC7753182

[98] Shahid M , Kim M , Jin P , Zhou B , Wang Y , Yang W , et al. S‐palmitoylation as a functional regulator of proteins associated with cisplatin resistance in bladder cancer. Int J Biol Sci. 2020;16:2490–505. 32792852 10.7150/ijbs.45640 PMC7415425

[99] Planey SL , Keay SK , Zhang CO , Zacharias DA . Palmitoylation of cytoskeleton associated protein 4 by DHHC2 regulates antiproliferative factor‐mediated signaling. Mol Biol Cell. 2009;20:1454–63. 19144824 10.1091/mbc.E08-08-0849 PMC2649263

[100] Yan SM , Tang JJ , Huang CY , Xi SY , Huang MY , Liang JZ , et al. Reduced expression of ZDHHC2 is associated with lymph node metastasis and poor prognosis in gastric adenocarcinoma. PLoS One. 2013;8:e56366. 23457560 10.1371/journal.pone.0056366 PMC3574152

[101] Sosa LDV , Petiti JP , Picech F , Chumpen S , Nicola JP , Perez P , et al. The ERα membrane pool modulates the proliferation of pituitary tumours. J Endocrinol. 2019;240:229–41. 30400032 10.1530/JOE-18-0418

[102] Pedram A , Razandi M , Deschenes RJ , Levin ER . DHHC‐7 and ‐21 are palmitoylacyltransferases for sex steroid receptors. Mol Biol Cell. 2012;23:188–99. 22031296 10.1091/mbc.E11-07-0638 PMC3248897

[103] Acconcia F , Ascenzi P , Bocedi A , Spisni E , Tomasi V , Trentalance A , et al. Palmitoylation‐dependent estrogen receptor alpha membrane localization: regulation by 17beta‐estradiol. Mol Biol Cell. 2005;16:231–7. 15496458 10.1091/mbc.E04-07-0547 PMC539167

[104] Cho E , Park M . Palmitoylation in Alzheimer's disease and other neurodegenerative diseases. Pharmacol Res. 2016;111:133–51. 27293050 10.1016/j.phrs.2016.06.008

[105] Yuan W , Lu LX , Rao MD , Huang Y , Liu CE , Liu S , et al. GFAP hyperpalmitoylation exacerbates astrogliosis and neurodegenerative pathology in PPT1‐deficient mice. Proc Natl Acad Sci USA. 2021;118:e2022261118. 33753498 10.1073/pnas.2022261118 PMC8020761

[106] Zhao L , Zhang C , Luo X , Wang P , Zhou W , Zhong S , et al. CD36 palmitoylation disrupts free fatty acid metabolism and promotes tissue inflammation in non‐alcoholic steatohepatitis. J Hepatol. 2018;69:705–17. 29705240 10.1016/j.jhep.2018.04.006

[107] Zhang M , Zhou L , Xu Y , Yang M , Xu Y , Komaniecki GP , et al. A STAT3 palmitoylation cycle promotes TH17 differentiation and colitis. Nature. 2020;586:434–9. 33029007 10.1038/s41586-020-2799-2 PMC7874492

[108] Lu Y , Zheng YP , Coyaud É , Zhang C , Selvabaskaran A , Yu YY , et al. Palmitoylation of NOD1 and NOD2 is required for bacterial sensing. Science. 2019;366:460–7. 31649195 10.1126/science.aau6391

[109] Wu Z , Zhang Z , Wang X , Zhang J , Ren C , Li Y , et al. Palmitoylation of SARS‐CoV‐2 S protein is essential for viral infectivity. Signal Transduct Target Ther. 2021;6:231. 34117209 10.1038/s41392-021-00651-y PMC8193602

[110] Woodley KT , Collins MO . S‐acylated Golga7b stabilises DHHC5 at the plasma membrane to regulate cell adhesion. EMBO Rep. 2019;20:e47472. 31402609 10.15252/embr.201847472 PMC6776912

[111] Aramsangtienchai P , Spiegelman NA , Cao J , Lin H . S‐palmitoylation of junctional adhesion molecule C regulates its tight junction localization and cell migration. J Biol Chem. 2017;292:5325–34. 28196865 10.1074/jbc.M116.730523 PMC5392678

[112] Heiler S , Mu W , Zoller M , Thuma F . The importance of claudin‐7 palmitoylation on membrane subdomain localization and metastasis‐promoting activities. Cell Commun Signal. 2015;13:29. 26054340 10.1186/s12964-015-0105-y PMC4459675

[113] Frohlich M , Dejanovic B , Kashkar H , Schwarz G , Nussberger S . S‐palmitoylation represents a novel mechanism regulating the mitochondrial targeting of BAX and initiation of apoptosis. Cell Death Dis. 2014;5:e1057. 24525733 10.1038/cddis.2014.17 PMC3944235

[114] Rebecca VW , Nicastri MC , Fennelly C , Chude CI , Barber‐Rotenberg JS , Ronghe A , et al. PPT1 promotes tumor growth and is the molecular target of chloroquine derivatives in cancer. Cancer Discov. 2019;9:220–9. 30442709 10.1158/2159-8290.CD-18-0706 PMC6368875

[115] Li Y , Martin BR , Cravatt BF , Hofmann SL . DHHC5 protein palmitoylates flotillin‐2 and is rapidly degraded on induction of neuronal differentiation in cultured cells. J Biol Chem. 2012;287:523–30. 22081607 10.1074/jbc.M111.306183 PMC3249106

[116] Bodin S , Planchon D , Rios Morris E , Comunale F , Gauthier‐Rouviere C . Flotillins in intercellular adhesion ‐ from cellular physiology to human diseases. J Cell Sci. 2014;127:5139–47. 25413346 10.1242/jcs.159764

[117] Carbonnelle D , Luu TH , Chaillou C , Huvelin JM , Bard JM , Nazih H . LXR activation down‐regulates lipid raft markers FLOT2 and DHHC5 in MCF‐7 breast cancer cells. Anticancer Res. 2017;37:4067–73. 28739689 10.21873/anticanres.11792

[118] Sharma C , Wang HX , Li Q , Knoblich K , Reisenbichler ES , Richardson AL , et al. Protein acyltransferase DHHC3 regulates breast tumor growth, oxidative stress, and senescence. Cancer Res. 2017;77:6880–90. 29055014 10.1158/0008-5472.CAN-17-1536 PMC5819883

[119] Sharma C , Yang W , Steen H , Freeman MR , Hemler ME . Antioxidant functions of DHHC3 suppress anti‐cancer drug activities. Cell Mol Life Sci. 2021;78:2341–53. 32986127 10.1007/s00018-020-03635-3 PMC8751980

[120] Stypulkowski E , Asangani IA , Witze ES . The depalmitoylase APT1 directs the asymmetric partitioning of Notch and Wnt signaling during cell division. Sci Signal. 2018;11:eaam8705. 29295957 10.1126/scisignal.aam8705 PMC5914505

[121] Berg V , Rusch M , Vartak N , Jungst C , Schauss A , Waldmann H , et al. miRs‐138 and ‐424 control palmitoylation‐dependent CD95‐mediated cell death by targeting acyl protein thioesterases 1 and 2 in CLL. Blood. 2015;125:2948–57. 25670628 10.1182/blood-2014-07-586511 PMC4654424

[122] Wang Y , Shi J , Chai K , Ying X , Zhou BP . The role of snail in EMT and tumorigenesis. Curr Cancer Drug Targets. 2013;13:963–72. 24168186 10.2174/15680096113136660102 PMC4004763

[123] Hernandez JL , Davda D , Majmudar JD , Won SJ , Prakash A , Choi AI , et al. Correlated S‐palmitoylation profiling of Snail‐induced epithelial to mesenchymal transition. Mol Biosyst. 2016;12:1799–808. 27030425 10.1039/c6mb00019c PMC5017304

[124] Sharma C , Rabinovitz I , Hemler ME . Palmitoylation by DHHC3 is critical for the function, expression, and stability of integrin alpha6beta4. Cell Mol Life Sci. 2012;69:2233–44. 22314500 10.1007/s00018-012-0924-6 PMC3406256

[125] Yeste‐Velasco M , Mao X , Grose R , Kudahetti SC , Lin D , Marzec J , et al. Identification of ZDHHC14 as a novel human tumour suppressor gene. J Pathol. 2014;232:566–77. 24407904 10.1002/path.4327

[126] Regan JL , Schumacher D , Staudte S , Steffen A , Haybaeck J , Keilholz U , et al. Non‐canonical hedgehog signaling is a positive regulator of the WNT pathway and is required for the survival of colon cancer stem cells. Cell Rep. 2017;21:2813–28. 29212028 10.1016/j.celrep.2017.11.025

[127] Dilly A , Honick BD , Lee YJ , Bartlett DL , Choudry HA . Rational application of targeted therapeutics in mucinous colon/appendix cancers with positive predictive factors. Cancer Med. 2020;9:1753–67. 31958897 10.1002/cam4.2847 PMC7050077

[128] Mansilla F , Birkenkamp‐Demtroder K , Kruhoffer M , Sorensen FB , Andersen CL , Laiho P , et al. Differential expression of DHHC9 in microsatellite stable and instable human colorectal cancer subgroups. Br J Cancer. 2007;96:1896–903. 17519897 10.1038/sj.bjc.6603818 PMC2359975

[129] Lu Y , Yan JS , Xia L , Qin K , Yin QQ , Xu HT , et al. 2‐Bromopalmitate targets retinoic acid receptor alpha and overcomes all‐trans retinoic acid resistance of acute promyelocytic leukemia. Haematologica. 2019;104:102–12. 30076181 10.3324/haematol.2018.191916 PMC6312026

[130] Krammer PH . CD95's deadly mission in the immune system. Nature. 2000;407:789–95. 11048730 10.1038/35037728

[131] Molica S , Mannella A , Dattilo A , Levato D , Iuliano F , Peta A , et al. Differential expression of BCL‐2 oncoprotein and Fas antigen on normal peripheral blood and leukemic bone marrow cells. A flow cytometric analysis. Haematologica. 1996;81:302–9. 8870373

[132] Remsberg JR , Suciu RM , Zambetti NA , Hanigan TW , Firestone AJ , Inguva A , et al. ABHD17 regulation of plasma membrane palmitoylation and N‐Ras‐dependent cancer growth. Nat Chem Biol. 2021;17:856–64. 33927411 10.1038/s41589-021-00785-8 PMC8900659

[133] Sharma G , Ojha R , Noguera‐Ortega E , Rebecca VW , Attanasio J , Liu S , et al. PPT1 inhibition enhances the antitumor activity of anti‐PD‐1 antibody in melanoma. JCI Insight. 2020;5:e133225. 32780726 10.1172/jci.insight.133225 PMC7526447

[134] Stolk DA , Horrevorts SK , Schetters STT , Kruijssen LJW , Duinkerken S , Keuning E , et al. Palmitoylated antigens for the induction of anti‐tumor CD8(+) T cells and enhanced tumor recognition. Mol Ther Oncolytics. 2021;21:315–28. 34141869 10.1016/j.omto.2021.04.009 PMC8170356

[135] Rebecca VW , Nicastri MC , McLaughlin N , Fennelly C , McAfee Q , Ronghe A , et al. A unified approach to targeting the lysosome's degradative and growth signaling roles. Cancer Discov. 2017;7:1266–83. 28899863 10.1158/2159-8290.CD-17-0741 PMC5833978

[136] Tian H , Lu JY , Shao C , Huffman KE , Carstens RM , Larsen JE , et al. Systematic siRNA screen unmasks NSCLC growth dependence by palmitoyltransferase DHHC5. Mol Cancer Res. 2015;13:784–94. 25573953 10.1158/1541-7786.MCR-14-0608 PMC4398612

[137] Mohammed A , Zhang C , Zhang S , Shen Q , Li J , Tang Z , et al. Inhibition of cell proliferation and migration in nonsmall cell lung cancer cells through the suppression of LYPLA1. Oncol Rep. 2019;41:973–80. 30431103 10.3892/or.2018.6857

[138] Chen X , Li H , Fan X , Zhao C , Ye K , Zhao Z , et al. Protein palmitoylation regulates cell survival by modulating XBP1 activity in glioblastoma multiforme. Mol Ther Oncolytics. 2020;17:518–30. 33024813 10.1016/j.omto.2020.05.007 PMC7525067

[139] Fan X , Yang H , Zhao C , Hu L , Wang D , Wang R , et al. Local anesthetics impair the growth and self‐renewal of glioblastoma stem cells by inhibiting ZDHHC15‐mediated GP130 palmitoylation. Stem Cell Res Ther. 2021;12:107. 33541421 10.1186/s13287-021-02175-2 PMC7863430

[140] Heuer TS , Ventura R , Mordec K , Lai J , Fridlib M , Buckley D , et al. FASN inhibition and Taxane treatment combine to enhance anti‐tumor efficacy in diverse xenograft tumor models through disruption of tubulin palmitoylation and microtubule organization and FASN inhibition‐mediated effects on oncogenic signaling and gene expression. EBioMedicine. 2017;16:51–62. 28159572 10.1016/j.ebiom.2016.12.012 PMC5474427

[141] Gruslova A , McClellan B , Balinda HU , Viswanadhapalli S , Alers V , Sareddy GR , et al. FASN inhibition as a potential treatment for endocrine‐resistant breast cancer. Breast Cancer Res Treat. 2021;187:375–86. 33893909 10.1007/s10549-021-06231-6

[142] Brun S , Bassissi F , Serdjebi C , Novello M , Tracz J , Autelitano F , et al. GNS561, a new lysosomotropic small molecule, for the treatment of intrahepatic cholangiocarcinoma. Invest New Drugs. 2019;37:1135–45. 30778887 10.1007/s10637-019-00741-3

[143] Brun S , Bestion E , Raymond E , Bassissi F , Jilkova ZM , Mezouar S , et al. GNS561, a clinical‐stage PPT1 inhibitor, is efficient against hepatocellular carcinoma via modulation of lysosomal functions. Autophagy. 2021;18:678–94. 34740311 10.1080/15548627.2021.1988357 PMC9037544

[144] Xu T , Huang C , Qi XT , Yang XC , Zhang N , Cao J , et al. 2‐Bromopalmitate sensitizes osteosarcoma cells to adriamycin‐induced apoptosis via the modulation of CHOP. Eur J Pharmacol. 2019;844:204–15. 30552901 10.1016/j.ejphar.2018.12.019

[145] Liu H , Yan P , Ren J , Wu C , Yuan W , Rao M , et al. Identifying the potential substrates of the depalmitoylation enzyme acyl‐protein thioesterase 1. Curr Mol Med. 2019;19:364–75. 30914023 10.2174/1566524019666190325143412

[146] Scifo E , Szwajda A , Soliymani R , Pezzini F , Bianchi M , Dapkunas A , et al. Proteomic analysis of the palmitoyl protein thioesterase 1 interactome in SH‐SY5Y human neuroblastoma cells. J Proteomics. 2015;123:42–53. 25865307 10.1016/j.jprot.2015.03.038

[147] Kong E , Peng S , Chandra G , Sarkar C , Zhang Z , Bagh MB , et al. Dynamic palmitoylation links cytosol‐membrane shuttling of acyl‐protein thioesterase‐1 and acyl‐protein thioesterase‐2 with that of proto‐oncogene H‐ras product and growth‐associated protein‐43. J Biol Chem. 2013;288:9112–25. 23396970 10.1074/jbc.M112.421073 PMC3610984

[148] Lin DT , Conibear E . ABHD17 proteins are novel protein depalmitoylases that regulate N‐Ras palmitate turnover and subcellular localization. Elife. 2015;4:e11306. 26701913 10.7554/eLife.11306 PMC4755737

[149] Lemonidis K , Salaun C , Kouskou M , Diez‐Ardanuy C , Chamberlain LH , Greaves J . Substrate selectivity in the zDHHC family of S‐acyltransferases. Biochem Soc Trans. 2017;45:751–8. 28620036 10.1042/BST20160309

[150] Davda D , El Azzouny MA , Tom CT , Hernandez JL , Majmudar JD , Kennedy RT , et al. Profiling targets of the irreversible palmitoylation inhibitor 2‐bromopalmitate. ACS Chem Biol. 2013;8:1912–7. 23844586 10.1021/cb400380s PMC3892994

[151] Vujic I , Sanlorenzo M , Esteve‐Puig R , Vujic M , Kwong A , Tsumura A , et al. Acyl protein thioesterase 1 and 2 (APT‐1, APT‐2) inhibitors palmostatin B, ML348 and ML349 have different effects on NRAS mutant melanoma cells. Oncotarget. 2016;7:7297–306. 26771141 10.18632/oncotarget.6907 PMC4872786

